# Supplementary material for: Speaking without vocal folds using a machine-learning-assisted wearable sensing-actuation system
Source: Nat Commun. 2024 Mar 12;15:1873. doi: 10.1038/s41467-024-45915-7 (PMC10933441; doi:10.1038/s41467-024-45915-7)
Supplement: Supplementary file 1 — Supplementary Information [file 41467_2024_45915_MOESM1_ESM.pdf]

Supplementary Information

Speaking without vocal folds using a machine-learning-assisted wearable sensing-actuation system

Ziyuan Che[1,2], Xiao Wan[1,2], Jing Xu[1], Chrystal Duan[1], Tianqi Zheng[1], Jun Chen[1,]*

[1]Department of Bioengineering, University of California, Los Angeles, Los Angeles, California 90095, USA

[2] These authors contributed equally to this work

*Correspondence to: jun.chen@ucla.edu (J. C.)

Table of Contents

Fig. S1. Stretchability of the MC layer with kirigami structure. 3
Fig. S2. Structural parameter of the device. 4
Fig. S3. Stress-strain curve of the kirigami structure. 5
Fig. S4. Unit design of the kirigami structure. 6
Fig. S5. Maximum strain with regards to the parameter of kirigami design. 7
Fig. S6. Horizontal isotropic stress layout of the device. 8
Fig. S7. Testing of horizontal sensing properties of the device. 9
Fig. S8. Vertical isotropic stress layout of the device. 10
Fig. S9. Testing of vertical sensing properties of the device. 11
Fig. S10. Response time, SNR and Sensitivity curve of the device under different stretching angles. 12
Fig. S11. Loudness of the device under different stretching (130%) angles. 13
Fig. S12. Current generated by different frequency and amplification (arb. units.) of the shaker. 14
Fig. S13. Sensing performance of the device with regards to different coil turn ratios. 15
Fig. S14. Sensing performance of the device with regards to concentrations of MC Layer. 16
Fig. S15. Sensing performance of the device with regards to PDMS ratios of the sensing membrane. 17
Fig. S16. Sensing performance of the device with regards to thickness of the sensing membrane. 18
Fig. S17. Sensing performance of the device with regards to thickness of the MC Layer. 19
Fig. S18. 3D Summary plots of the optimizing parameters' influence on Response time, SNR, and Sensitivity 20
Fig. S19. Durability of the device. 21
Fig. S20. Loudness of the actuation component at different angles. 22
Fig. S21. Loudness of each resonance points at different strains. 23
Fig. S22. Sound pressure level across whole human hearing range under different strain. 24
Fig. S23. Right shift of first resonance point of each strain. 25
Fig. S24. Sound pressure level of the device under different coil turn ratios. 26
Fig. S25. Sound pressure level of the device under different PDMS ratio of actuator membrane. 27
Fig. S26. Sound pressure level of the device under different magnetic powder concentrations. 28
Fig. S27. Sound pressure level of the device under different MC layer thickness. 29
Fig. S28. Sound pressure level of the device under different actuator membrane thickness. 30
Fig. S29. Waveform and spectrogram of the device under 115% and 130% strains. 31
Fig. S30. Waveform the device under water and in air. 32
Fig. S31. Spectrogram of the device under water and in air. 33
Fig. S32. Accelerated aging test of the device for waterproofness. 34
Fig. S33. Sound pressure level of the device at different depth under water. 35
Fig. S34. Sound pressure level of the device in air (yellow) and under water (red). 36
Fig. S35. Confusion matrix of each participant's validation set (blue) and testing set (green). 37
Supplementary Notes 1: Fabrication and performance of the serpentine-shaped coil. 37
Supplementary Notes 2: Deformation of the device under omnidirectional laryngeal movements 41
Supplementary Notes 3: Examination of the output current and exclusion of triboelectricity 43
Supplementary Table S1. Weight of different parts of the device 44
Supplementary Table S2. Thickness of the coils with different turn ratios 45
Supplementary Table S3. Acoustic performance comparison with other literature 46
Supplementary References 47

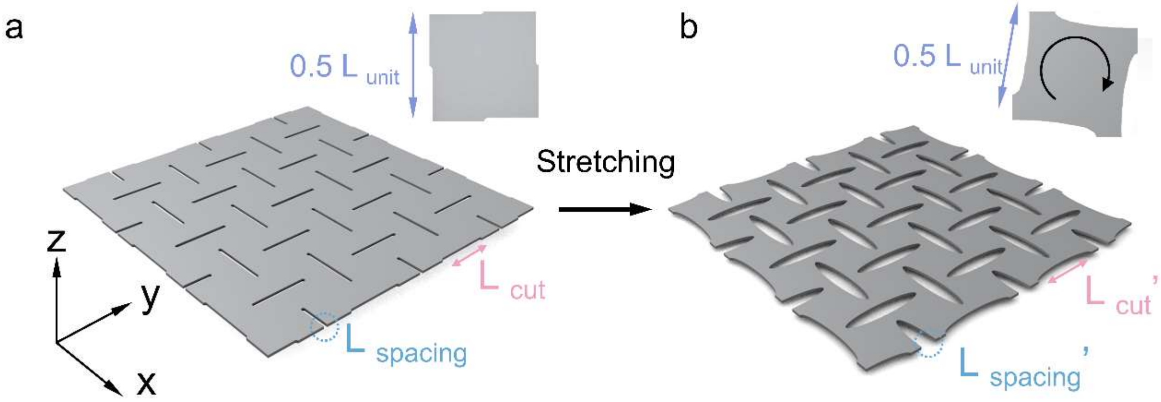

Fig. S1 | Stretchability of the MC layer with a kirigami structure. Kirigami-structured MC layer demonstrated improved stretchability and strain distribution. The inset figure depicts a single unit of the structure, exhibiting rotation and enlarged $L_{spacing}$ and L_{cut} upon stretching, as seen in the before-after images. **a**, illustration of the device before stretching. **b**, illustration of the device after stretching.

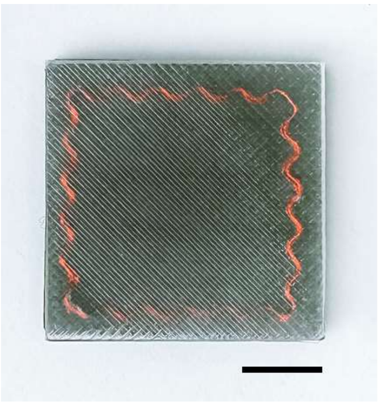

Fig. S2 | Structural parameter of the device. As shown in the figure, the MC layer is squarely shaped with a side length of 30mm. 12 half-circle-shaped units with 2.16mm diameter in the serpentine copper coil, spanning 25.92mm, scale bar, 10mm.

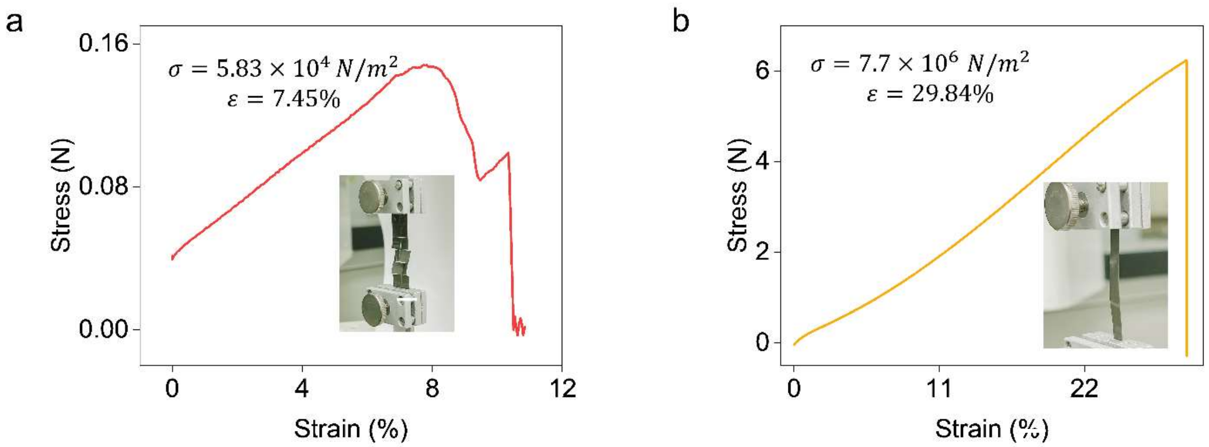

Fig. S3 | Stress-strain curve of the kirigami structure. a, the stress and strain curve of the MC layer fabricated with kirigami structure, insect figure shows the testing photo, scale bar 5mm. The testing results in a maximum strain of 7.45% with a maximum stress of $5.83 \times 10^4\ N/m^2$. The Young's modulus is calculated to be 7.83×10^5 Pa. **b**, the stress and strain curve of the MC layer without the kirigami structure, insect figure shows the testing photo, scale bar 5mm. The testing results in a maximum strain of 29.84% with a maximum stress of $7.7 \times 10^6\ N/m^2$, resulting in a higher young's modulus of 2.589×10^7 Pa.

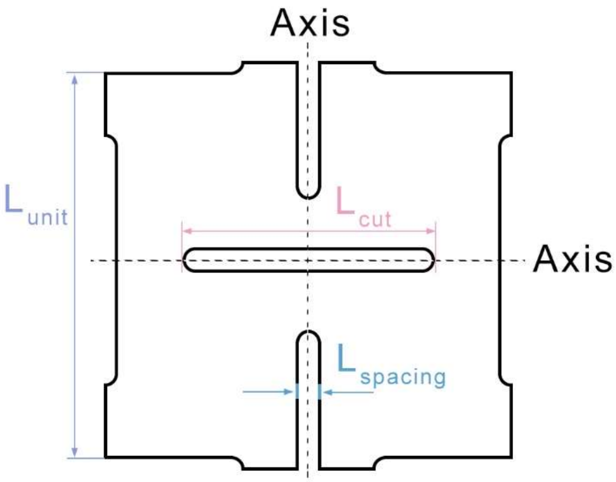

Fig. S4 | Unit design of the kirigami structure. The unit design of the kirigami structure is a square-shaped axisymmetric pattern, the two symmetric axes are illustrated in the figure. Three main parameters determine the pattern of the kirigami design: $L_{spacing}$, L_{cut}, and L_{unit}. L_{unit} in our design is determined by the entire size of the device (30 mm) to be 6mm. $L_{spacing}$ is optimized by the performance of laser cutter of 1mm.

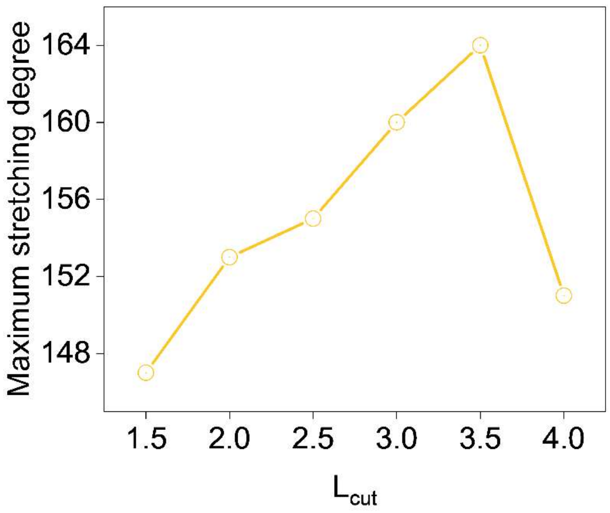

Fig. S5 | Maximum strain with regards to the parameter of kirigami design. The relationship between parameter L_{cut} in the unit design and the maximum strain. As shown in the figure above, with gradual increase of the length of L_{cut}, the stretchability increases since larger gaps can be created during stretching. However, the structure tends to break at the edge of the cut when L_{cut} is set too long. With that, the final parameter is determined at 3.5mm.

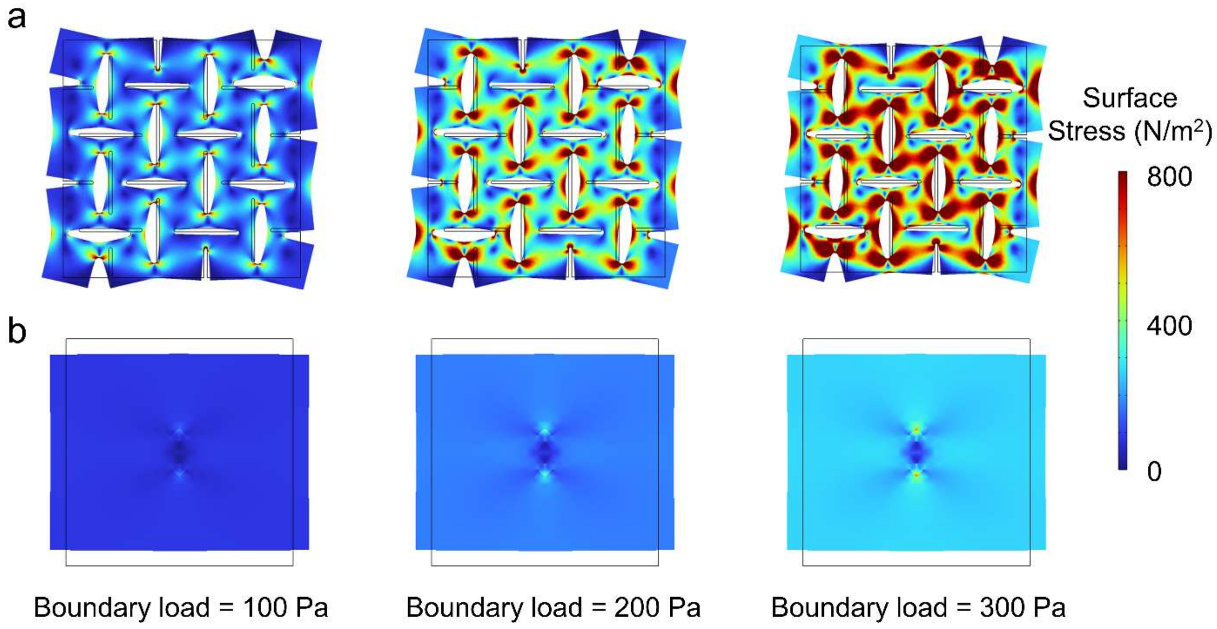

Fig. S6 | Horizontal isotropic stress layout of the device. (**a**) and (**b**) present COMSOL simulation results of a kirigami design and a layer without kirigami design, respectively, under varying boundary stress in the horizontal direction. The kirigami design exhibits an even stress distribution with localized concentration at the cut edges, enhancing resistance to random and uneven body movements during use. This feature eliminates specific wearing orientation requirements, improving user-friendliness. Notably, the kirigami design induces more horizontal deformation than the non-kirigami layer under identical boundary stress, as depicted in the figures.

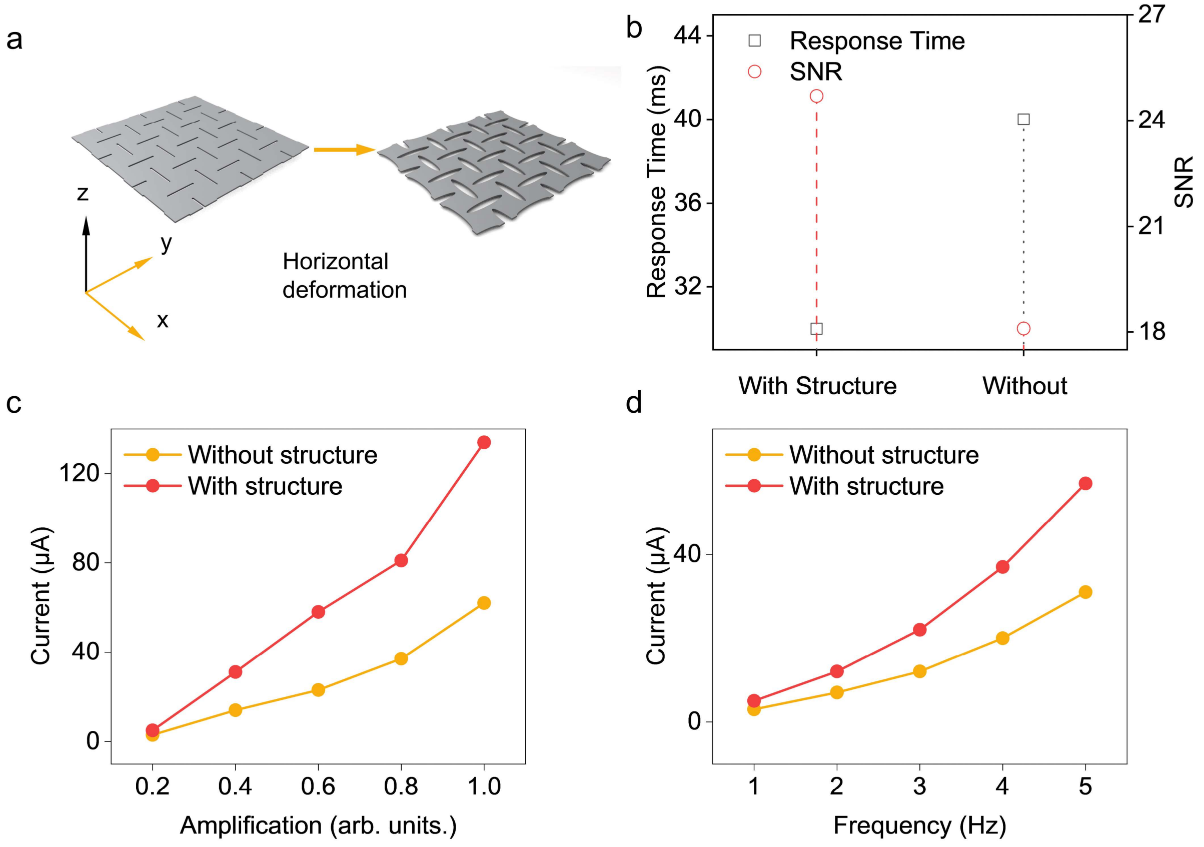

Fig. S7 | Testing of horizontal sensing properties of the device. a, schematic illustration of horizontal deformation, the following sensing properties are tested with deformation happening in the x and y axis without direct stress on the vertical (z-axis) direction. **b**, Response time and signal to noise ratio of the device with and without the fabrication of kirigami structure. The structure design elevates the SNR (from 18.1 to 24.7) and lowers the response time by 10 ms (40 – 30 ms). **c**, the sensitivity curve of the device under different amplification of the shaker, the higher the amplification, the larger the deformation. The kirigami structure enlarges the deformation under same stress and consequently increases the unit magnetic flux change. Thus, creating a higher response in current and higher sensitivity of the device to muscle stretching. (arb. units.) referring to arbitrary units. **d**, the sensitivity curve of the device under different frequencies of the shaker. Since the muscle movement is a low-frequency signal, a gratitude of frequency from 1-5 Hz are tested. The higher the frequency, faster the deformation happens and that will generate a higher current signal. The kirigami structural design creates a higher sensitivity to low frequency signals with larger deformation under the same stress.

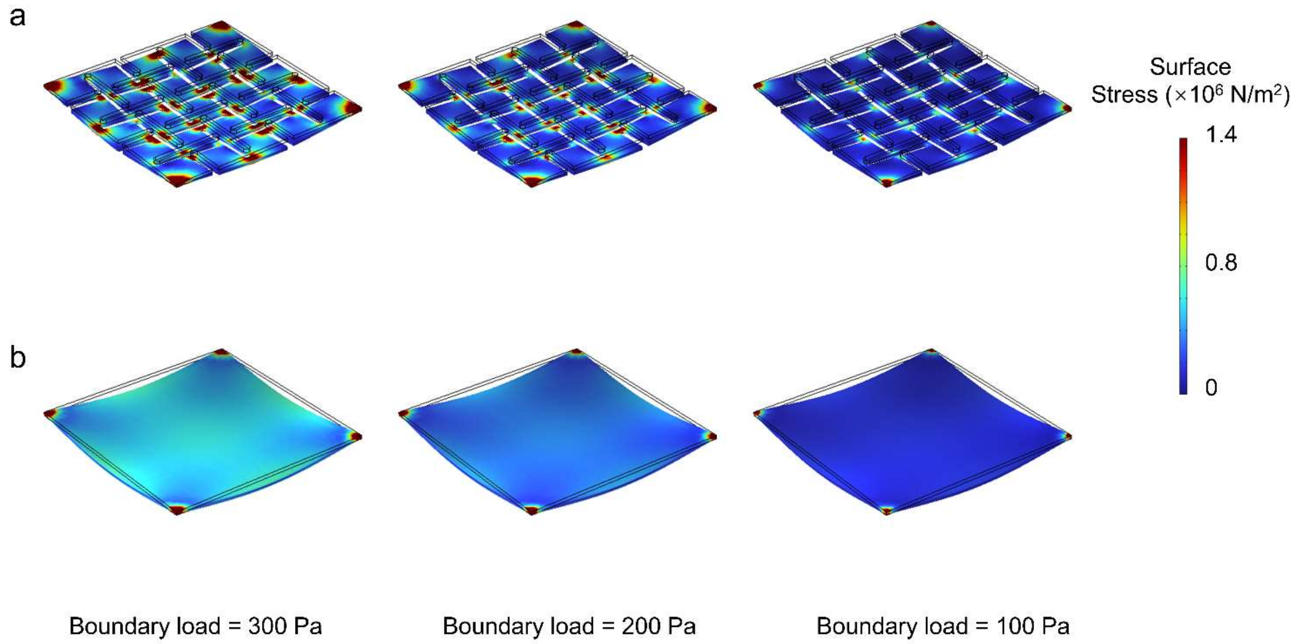

Fig. S8 | Vertical isotropic stress layout of the device. a, The COMSOL simulation results of the kirigami design under different boundary stress (vertical direction). **b**, The COMSOL simulation results of the layer without kirigami design under different boundary stress (vertical direction). As shown in **a**, the stress is spread evenly throughout the layer with a local focus at the edge of the cut. This stress layout pushes the device to a greater deformation at the cut of the kirigami structure, increasing the deformation in the vertical direction and elevates the detection sensitivity of vertical muscle movement.

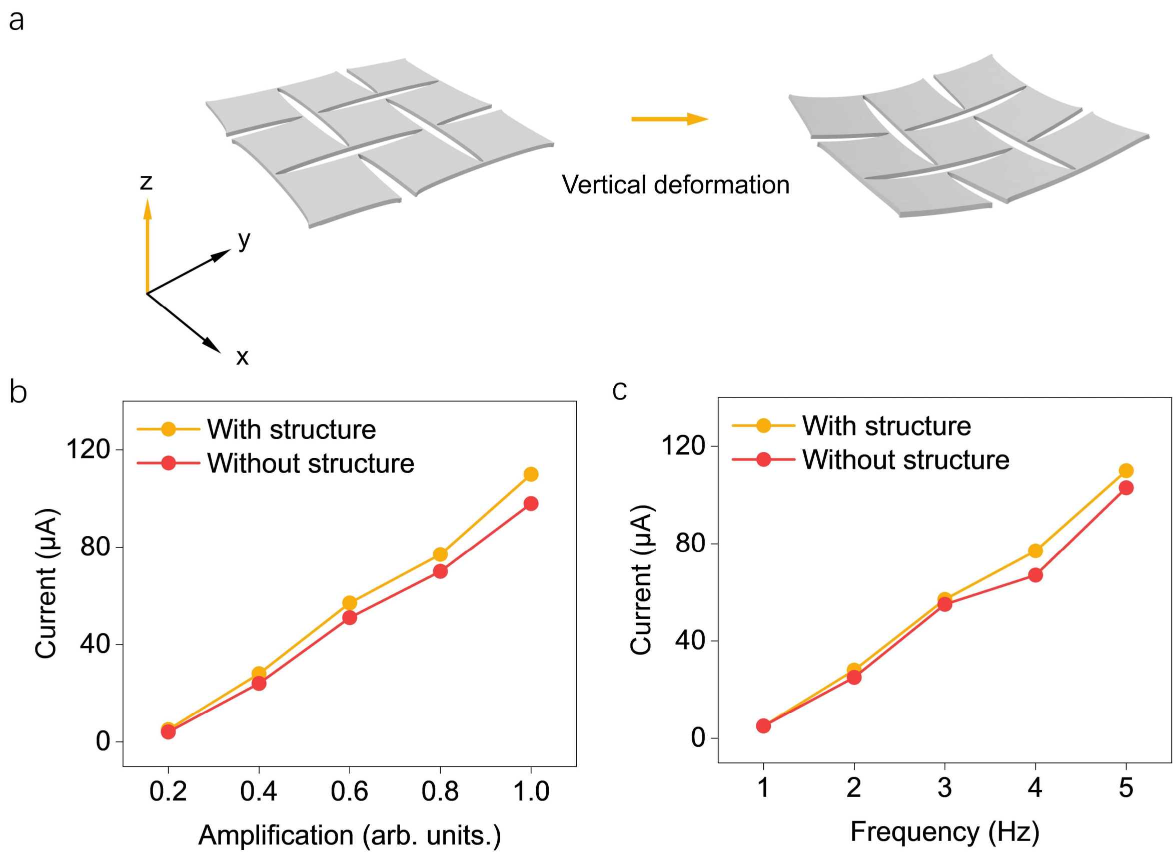

Fig. S9 | Testing of vertical sensing properties of the device. a, schematic illustration of vertical deformation, the following sensing properties are tested with deformation happening in the z-axis without direct stress on the horizontal (x, y-axis) direction. **b**, the sensitivity curve of the device under different amplification of the shaker, the higher the amplification, the larger the deformation. The kirigami structure enlarges the deformation under same stress and consequently increases the unit magnetic flux change. Thus, creating a higher response in current and higher sensitivity of the device to muscle vibration. (arb. units.) referring to arbitrary units. **c**, the sensitivity curve of the device under different frequencies of the shaker. Since the muscle movement is a low-frequency signal, a gratitude of frequency from 1-5 Hz are tested. The higher the frequency, faster the deformation happens and that will generate a higher current signal. The kirigami structural design creates a higher sensitivity to low frequency signals with larger deformation under the same stress.

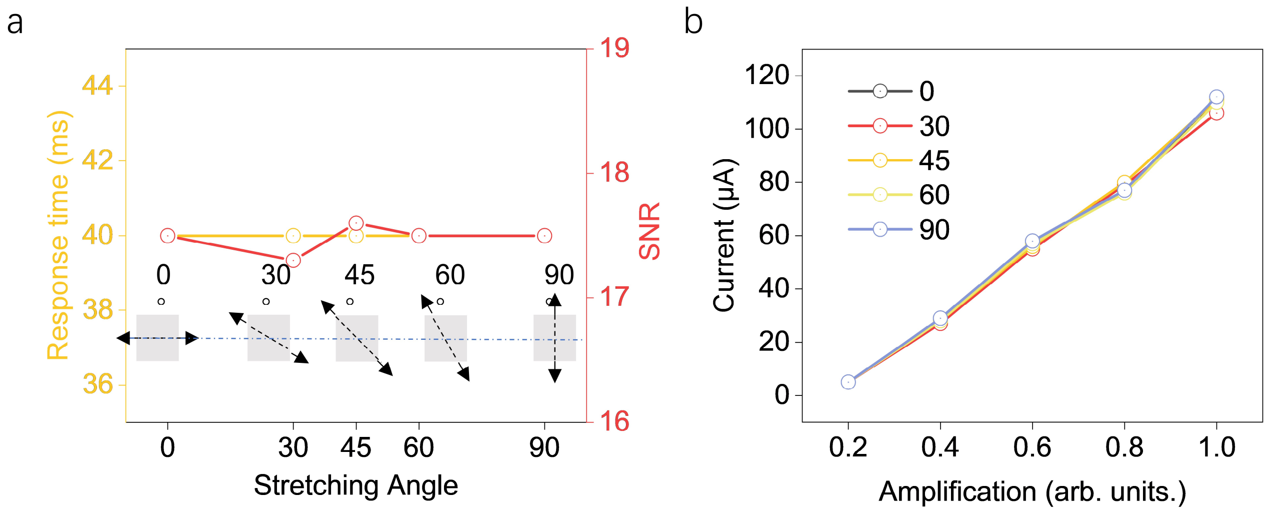

Fig. S10 | Response time, SNR and Sensitivity curve of the device under different stretching (130%) angles. **a**, The response time and Signal-Noise ratio with regards to different stretching angles under 130% strains. The response time has no change and a slight fluctuation of SNR is observed as the stretching angle changes. **b**, the sensitivity curve under different stretching angles. No obvious difference can be discerned, indicating the irrelevance between stretching angles and the sensing properties of the device. (arb. units.) referring to arbitrary units.

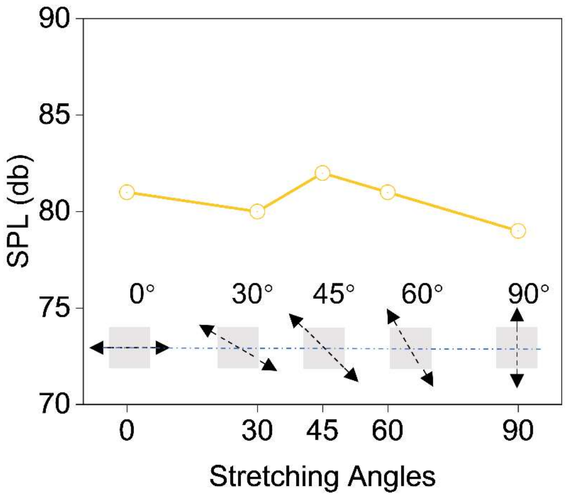

Fig. S11 | Loudness of the device under different stretching (130%) angles. The sound pressure level output by the device (a tone of 1000 Hz) regarding the stretching angles at a uniform strain of 130%. Only a slight fluctuation around 80 dB can be observed, indicating negligible influence of stretching angles on sound output of the device.

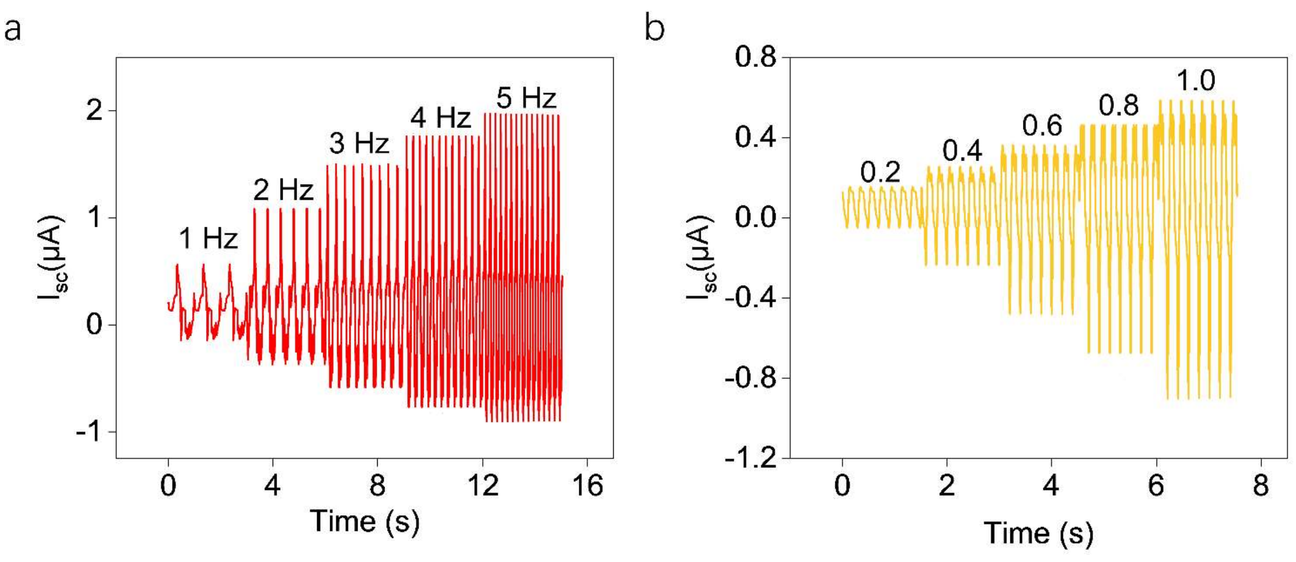

Fig. S12 | Current generated by different frequency and amplification (arb. units.) of the shaker. a, The current generated by the sensor at different frequencies of the shaker. With higher frequency, the number and amplitude of the peaks generated get larger as the shaker moves quicker under high frequency and induces faster change of the magnetic flux. **b**, The current generated by the sensor at different amplification of the shaker with the same frequency. Without the number changes, the amplitude of the peak enlarges as the amplification of the shaker increases. Conclusively, the sensor we developed can respond to input with different strength and frequency and generate corresponding signals.

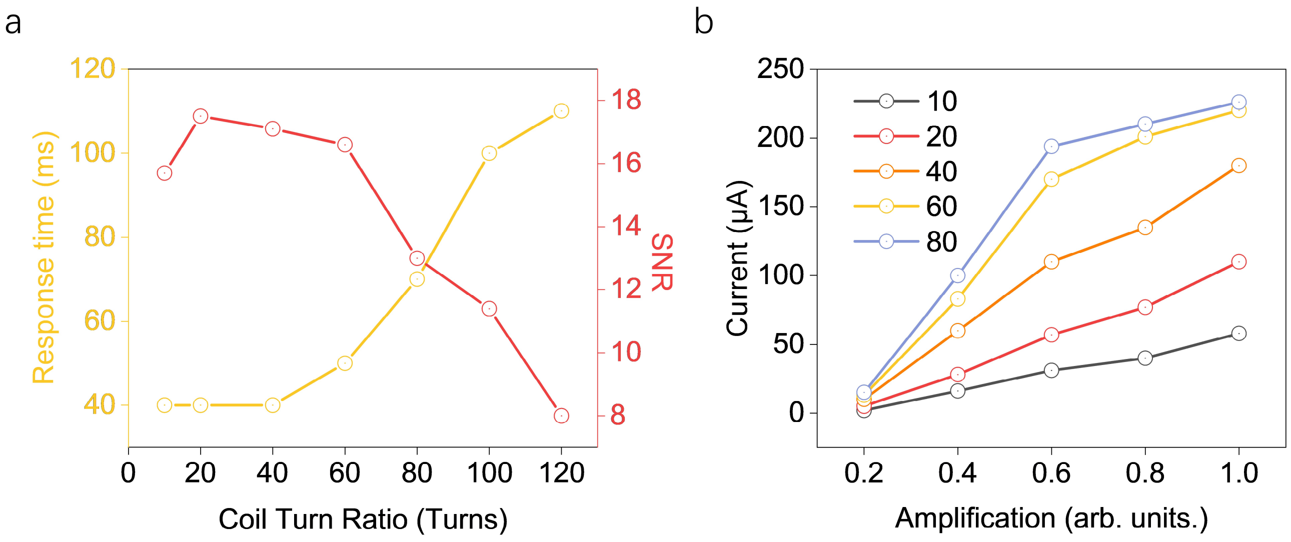

Fig. S13 | Response time, signal-to-noise ratio (SNR) and sensitivity of the device with regards to different coil turn ratios. a, the response time and Signal-Noise ratio regarding coil turns. More coil turns results in longer response times and lower SNR as the total thickness of the copper coil increases (especially after 40 turns). Consequently, the thickness of the coil will hinder the deformation of the membrane during vibrations, causing longer response time and lower signal quality. **b**, the sensitivity curve under different coil turns. Similarly, the thickness of the coil hinders the linearity as the coil turn increases. Based on both results, we have determined the turns to be 20 in our design. (arb. units.) referring to arbitrary units.

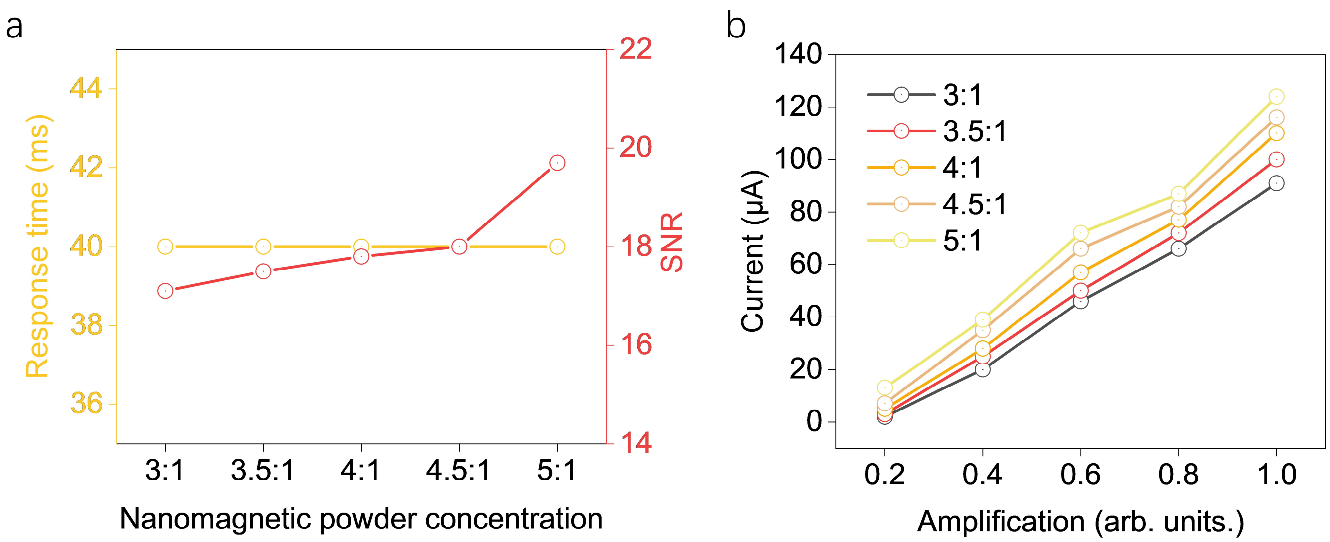

Fig. S14 | Response time, SNR, and sensitivity of the device with regards to different nanomagnetic powder concentrations of MC Layer. a, the response time and Signal-Noise ratio regarding nanomagnetic powder concentrations. No changes in response time and a slight increase in SNR is observed as the concentration of nanomagnetic powder gets larger. **b**, the sensitivity curve under different nanomagnetic powder concentrations. A semi-linear relationship can be observed as under each amplification, higher nanomagnetic powder concentration will generate stronger magnetic field and consequently generate higher current output. While this increase does not change the sensitivity (relationship among different amplifications) of the device. (arb. units.) referring to arbitrary units.

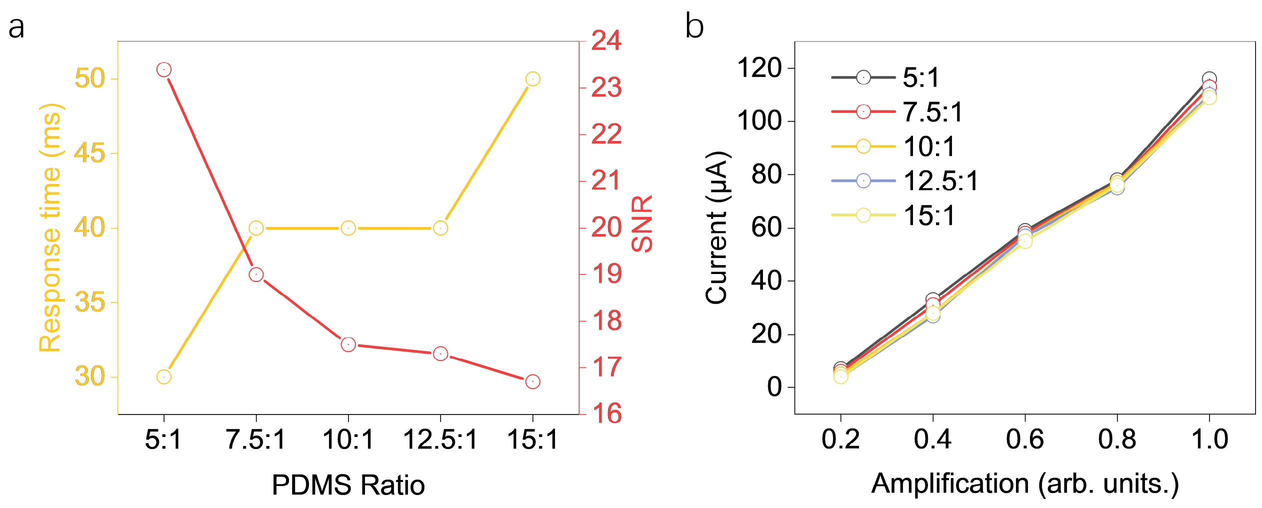

Fig. S15 | Response time, SNR, and sensitivity of the device with regards to different PDMS ratios of the sensing membrane. a, the response time and SNR regarding PDMS Ratios (the ratio between polymer base and curing agent). A higher ratio results in longer response times and lower SNR. **b**, the sensitivity curve under different PDMS ratios. Almost no change in the sensitivity curve has been caused by the PDMS ratio. This might be because the deformation caused by the shaker is large enough to overcome the difference in harness under different ratios. While this change in hardness does have an influence on response time and SNR since harder membrane (less ratio) tends to have quicker response time (the deformation happens quicker) and higher signal quality (the renounce of the deformation is less). However, lower ration with higher hardness changes the young's modulus of the device and reduces wearing comfortability. Here we choose the ration of 10:1 as a compromising point between sensing performance and wearing comfort. (arb. units.) referring to arbitrary units.

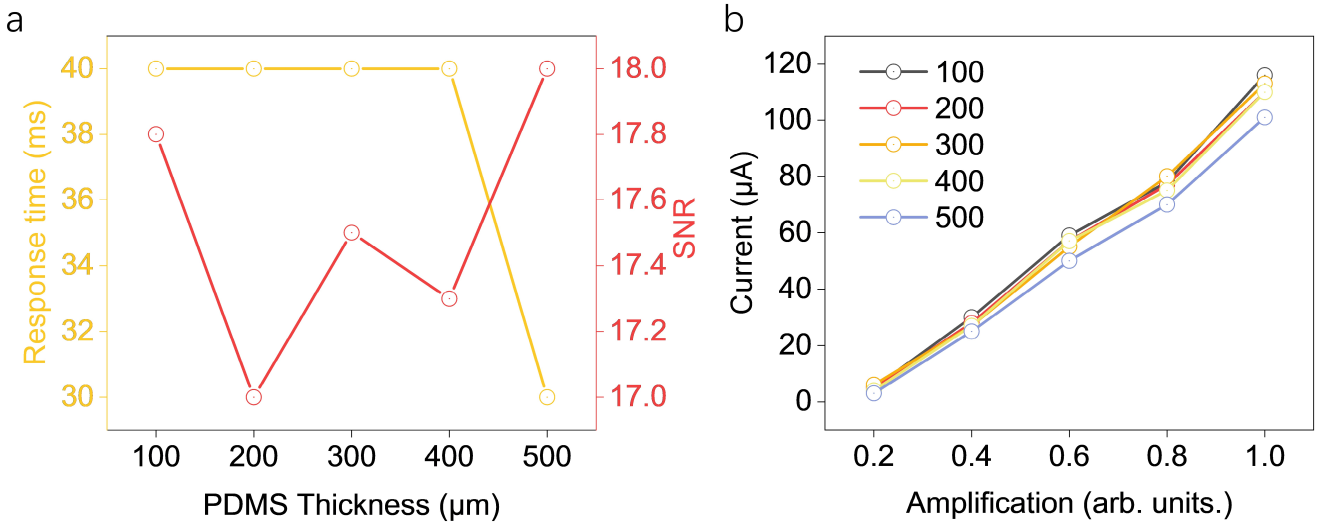

Fig. S16 | Response time, SNR, and sensitivity of the device with regards to different thickness of the sensing membrane. a, the response time and SNR regarding PDMS thickness. The thicker membrane results in quicker response times and a fluctuating SNR. **b**, the sensitivity under different PDMS ratios. Only a slight decrease in the sensitivity has been caused by the PDMS thickness. With the results, we have determined the thickness in our design to be 200 μm. Even though higher thickness has quicker response time, the thicker membrane will affect the wearing comfort and adherence of the device attached to the skin. (arb. units.) referring to arbitrary units.

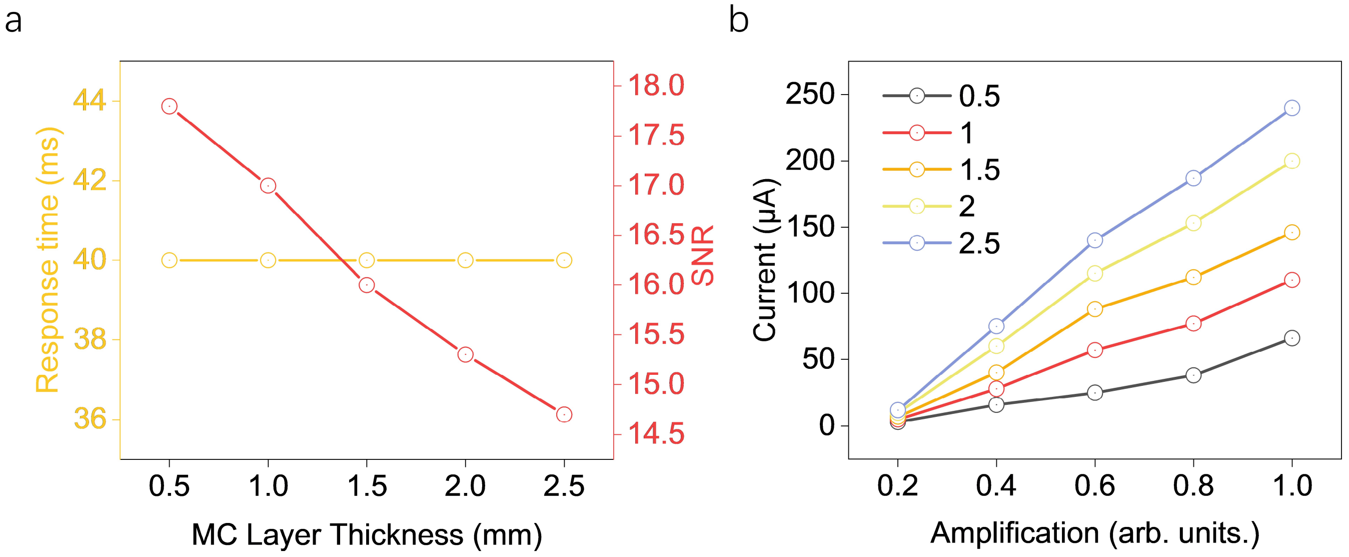

Fig. S17 | Response time, SNR, and sensitivity of the device with regards to different thickness of the MC Layer. **a**, the response time and SNR regarding the PDMS thickness. Thicker MC layer has no influence on response time while reduces SNR. **b**, the sensitivity under different MC layer thickness. An even increase in the sensitivity can be observed. Since thicker MC layer creates stronger magnetic field, the current generated at each amplification level is larger. Similarly, the noise signal is also stronger, thus, reducing the SNR. (arb. units.) referring to arbitrary units.

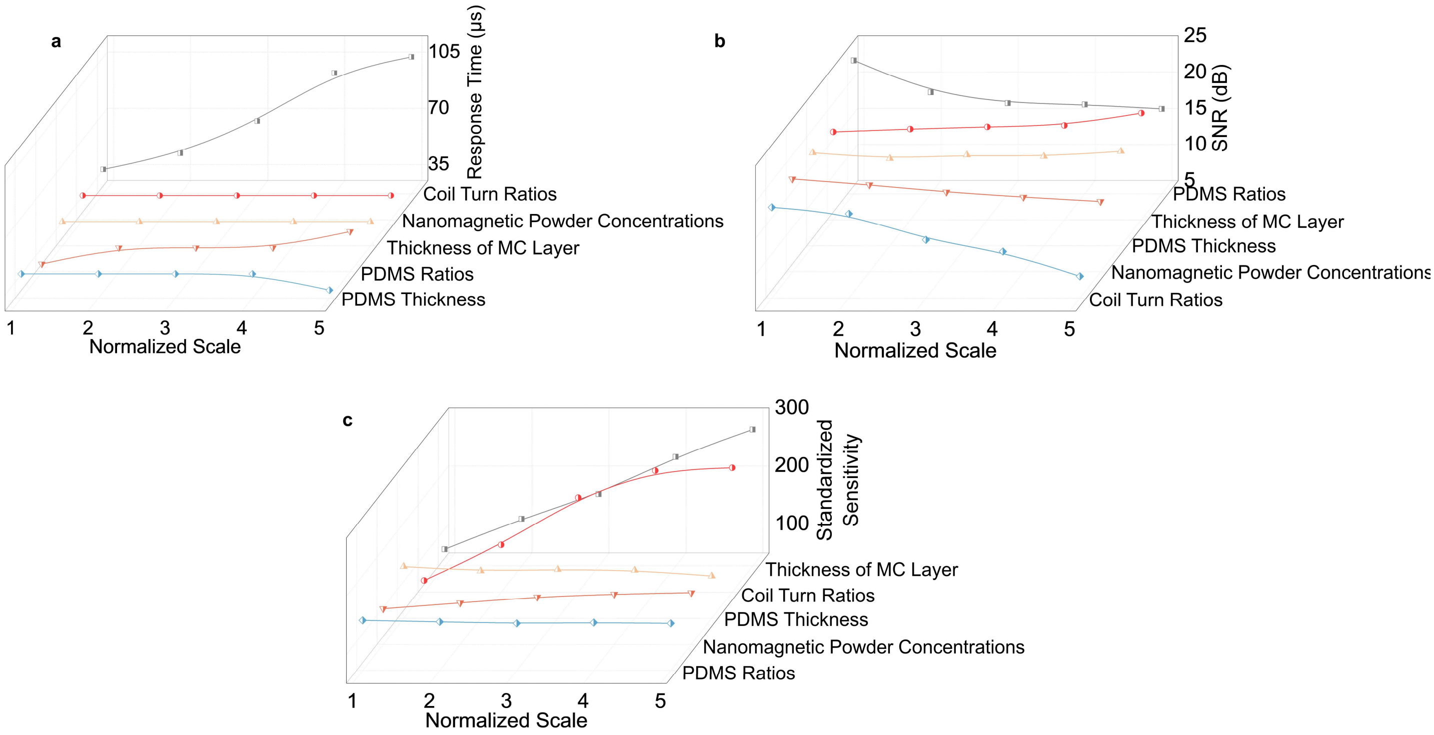

Fig. S18 | 3D Summary plots of the optimizing parameters' influence on Response time, SNR, and Sensitivity. a, Response time of the device as influenced by coil turns, MC layer thickness (μm), nanomagnetic powder concentration, MI layer thickness (μm), and PDMS ratio. **b**, SNR of the device with variations in coil turns, MC layer thickness (μm), nanomagnetic powder concentration, MI layer thickness (μm), and PDMS ratio. **c**, Sensitivity of the device in relation to coil turns, MC layer thickness (μm), nanomagnetic powder concentration, MI layer thickness (μm), and PDMS ratio.

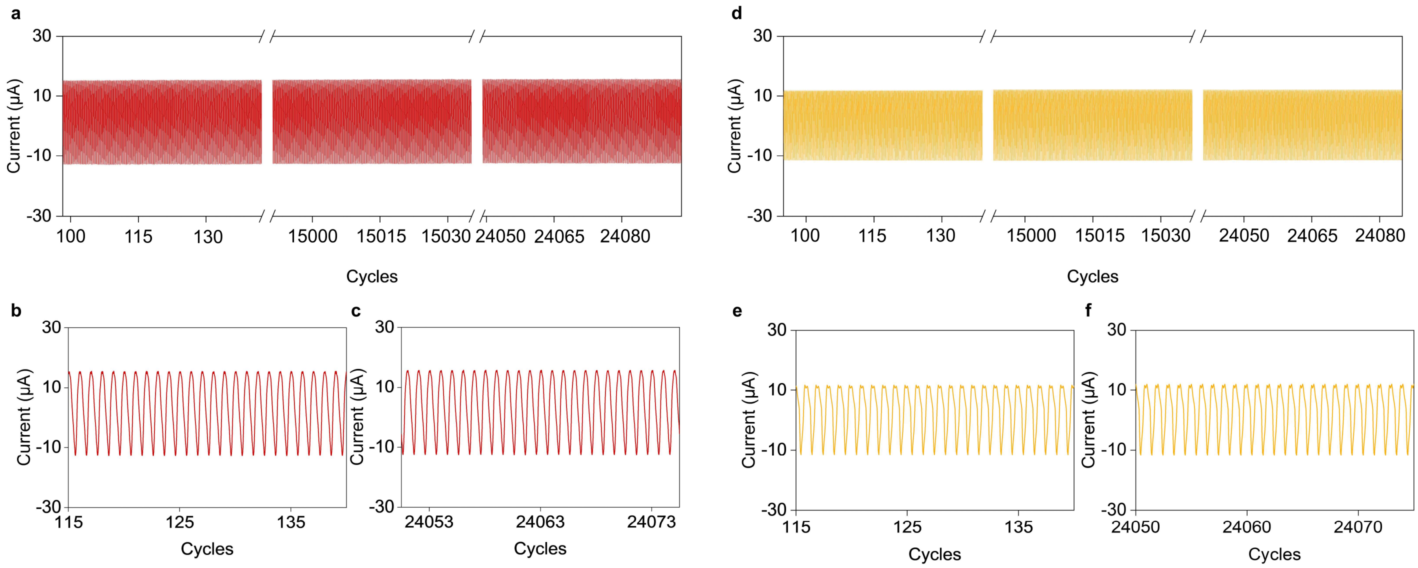

Fig. S19 | Durability of the device. The durability of the device was evaluated by continuously working for 24,000 cycles *via* a shaker at a frequency of 5 Hz. **a,** the overall waveform of the device tested without stretching from 1-24,000 cycles. **b,** Enlarged view of the 115-140 cycles of the device. **c**, enlarged view of the 24050-24075 cycles of the device. **d,** the overall waveform of the device tested under 135% strain from 1-24,000 cycles. **e**, enlarged view of the 115-140 cycles of the stretched device. **f**, enlarged view of the 24050-24075 cycles of the stretched device.

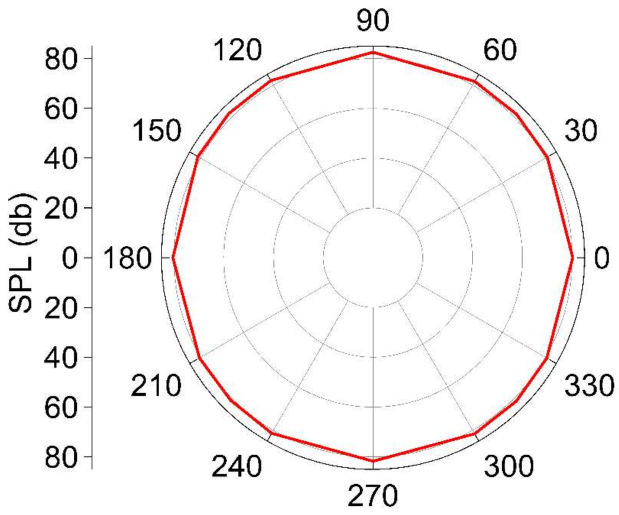

Fig. S20 | Loudness of the actuation component at different angles. The sound pressure level output by the actuation component at different angles (with a tone of 1000 Hz). An even performance (slight fluctuating around 80dB) across all angles can be observed.

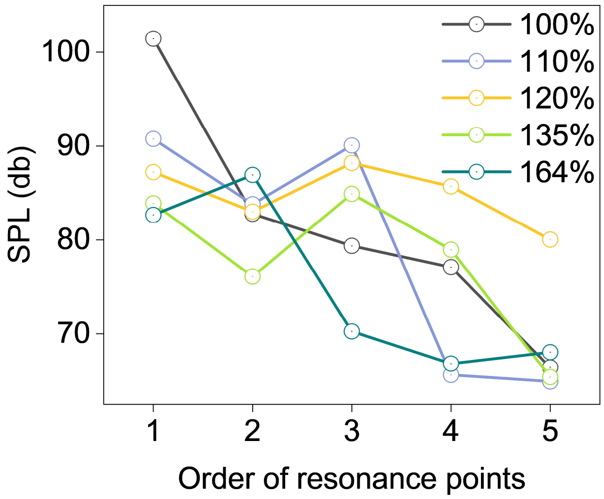

Fig. S21 | Loudness of each resonance points at different strains. The sound pressure level of each resonance points under different strains. Under most strains, the first resonance point is the point of largest sound pressure level. At the largest strain of 164% the loudest resonance point is the second one, but the first point has an only slightly lower sound pressure level (SPL) (less than 5dB).

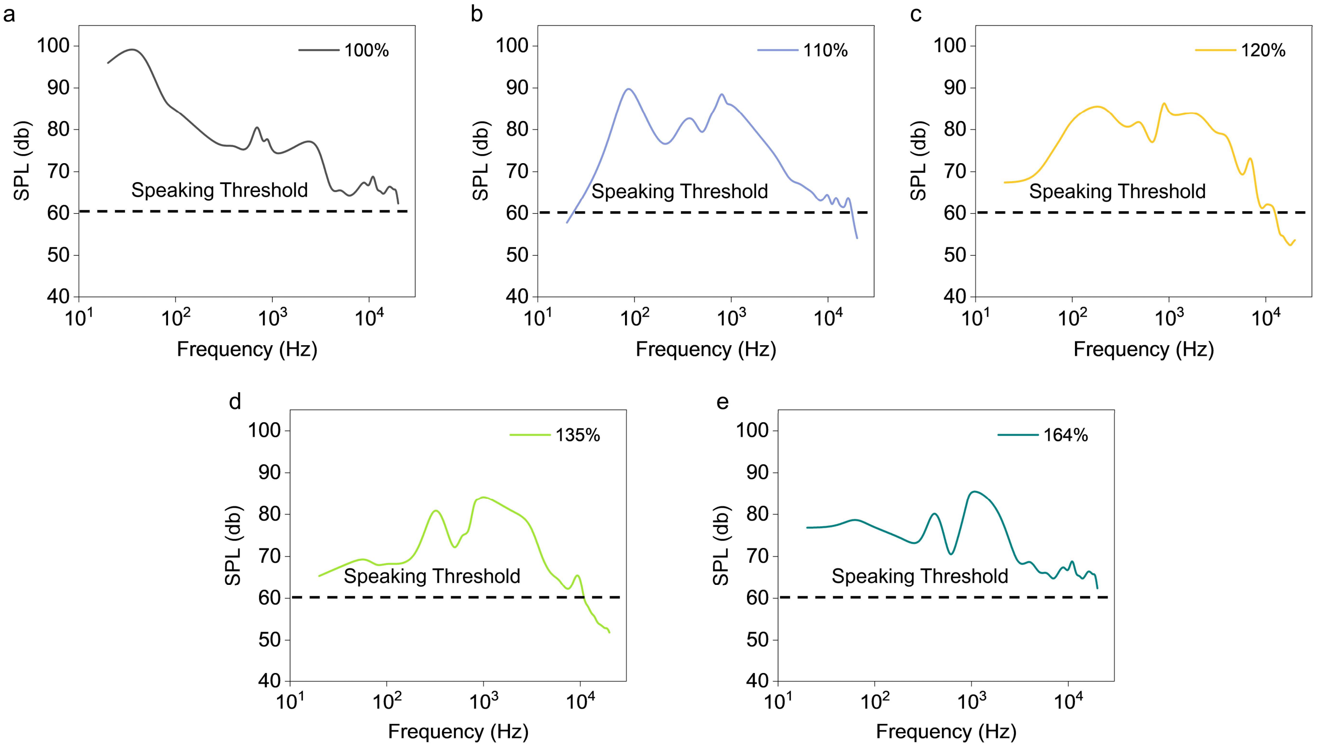

Fig. S22 | Sound pressure level across whole human hearing range under different strains. a, Sound pressure level of the device under a strain of 100% (no stretching) at the frequency of 20-20,000 Hz. **b,** Sound pressure level of the device under a strain of 110% at the frequency of 20-20,000 Hz. **c,** Sound pressure level of the device under a strain of 120% at the frequency of 20-20,000 Hz. **d,** Sound pressure level of the device under a strain of 135% at the frequency of 20-20,000 Hz. **e,** Sound pressure level of the device under a strain of 164% at the frequency of 20-20,000 Hz. All outputs are larger than the threshold of normal human speaking (40 – 60 dB) across the whole human hearing frequency range.

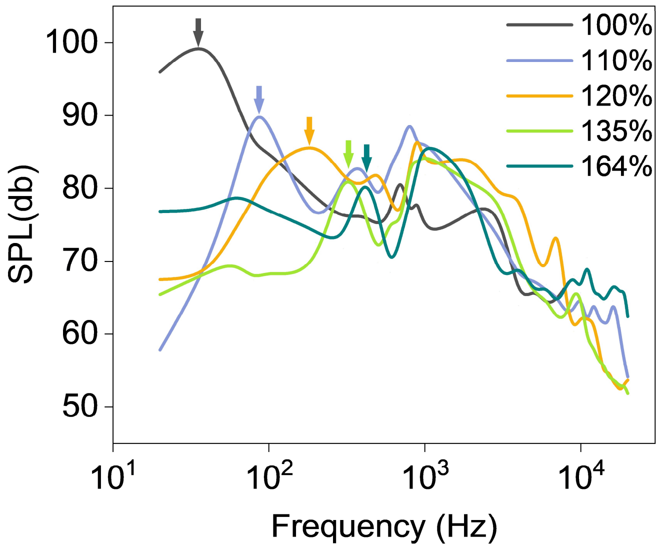

Fig. S23 | Right shift of first resonance point of each strain. The assembled graph of SPL-Frequency under each strain, the first resonance point is pointed out with arrow of corresponding colors. A clear right shift towards higher frequency can be observed, the larger the strains, the higher the frequency the first resonance point lies.

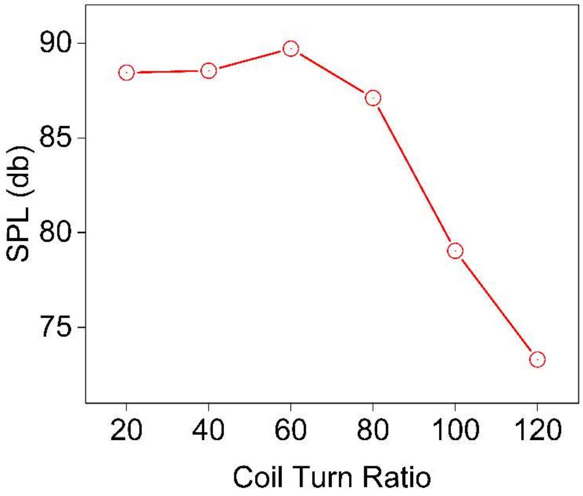

Fig. S24 | Sound pressure level of the device under different coil turn ratios. The relationship between coil turn ratio and sound pressure level (generated at a frequency of 1000 Hz), with more turns of coil, the SPL generated get lower. The weight of large amounts of coil hinders the vibration of the membrane and further reduces the SPL.

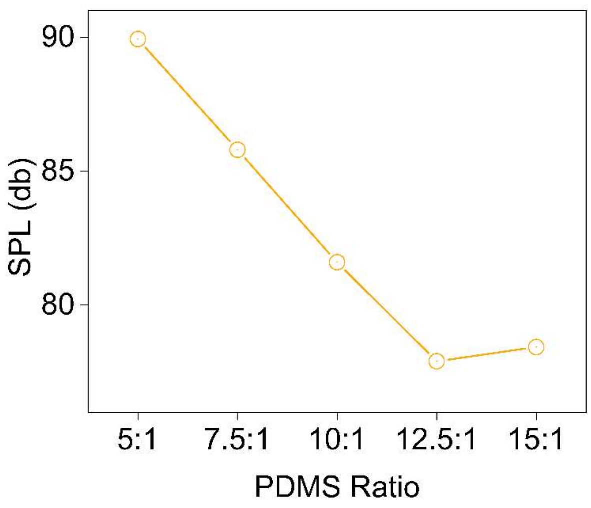

Fig. S25 | Sound pressure level of the device under different PDMS ratio of actuator membrane. The relationship between PDMS ratio (the ratio between polymer base and curing agent) and sound pressure level (generated at a frequency of 1000 Hz), as the ratio gets larger the membrane will be harder and subsequently the sound pressure level generated will be lower. The dampen effect of softer membrane will hinder the vibration and the generation of sounds, thus a semi-linear decrease can be observed.

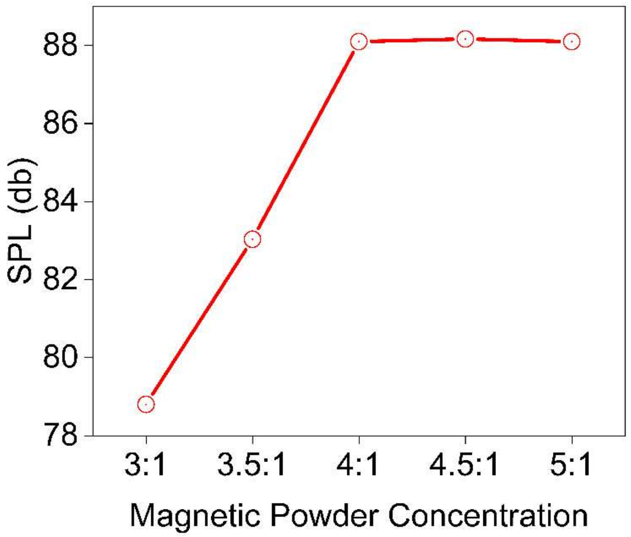

Fig. S26 | Sound pressure level of the device under different magnetic powder concentrations. The sound pressure level of the device elevates as a higher amount of magnetic powder (in the MC layer) is added and then plateaus after the ratio of 4:1.

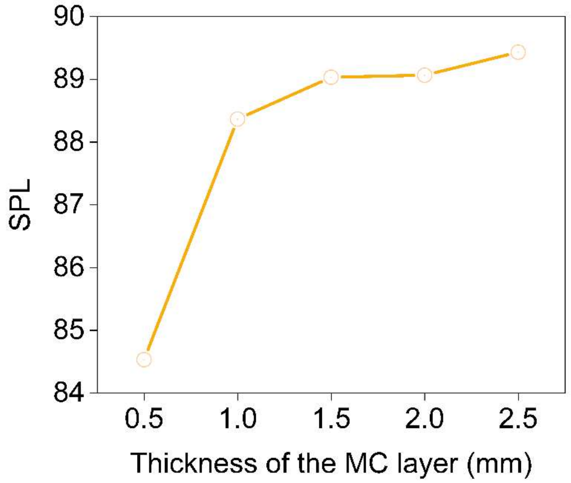

Fig. S27 | Sound pressure level of the device under different MC layer thickness. A sharp increase in sound pressure level generated by the device can be obtained as the thickness of the MC layer increases from 0.5 mm to 1 mm. Afterwards the increase slows downs and eventually plateaus as the MC layer gets even thicker.

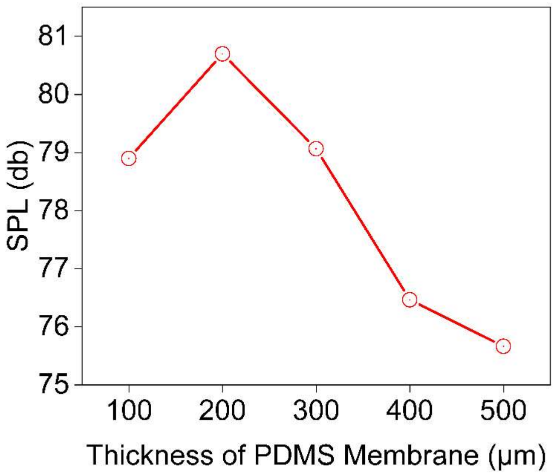

Fig. S28 | Sound pressure level of the device under different actuator membrane thickness. The sound pressure level of the device gets louder as the PDMS membrane (vibrating membrane) gets thicker from 100 μm to 200 μm and then reduces when the membrane turns thicker. The weight of thicker membranes might dampen the vibration and reduce the loudness produced by the device.

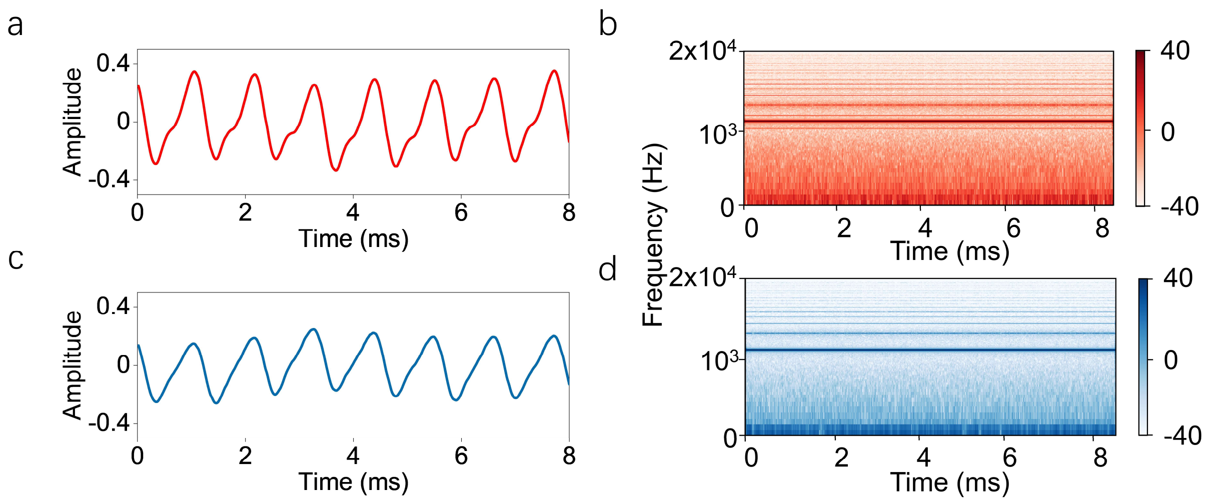

Fig. S29 | Waveform and spectrogram (1100 Hz) of the device under 115% (blue) and 130% (red) strains. **a,** the wave form of sound generated by the device at 130% strains. **b**, the spectrogram of the sound generated by the device at 130% strains. **c**, the wave form of sound generated by the device at 115% strains. **d**, the spectrogram of the sound generated by the device at 115% strains.

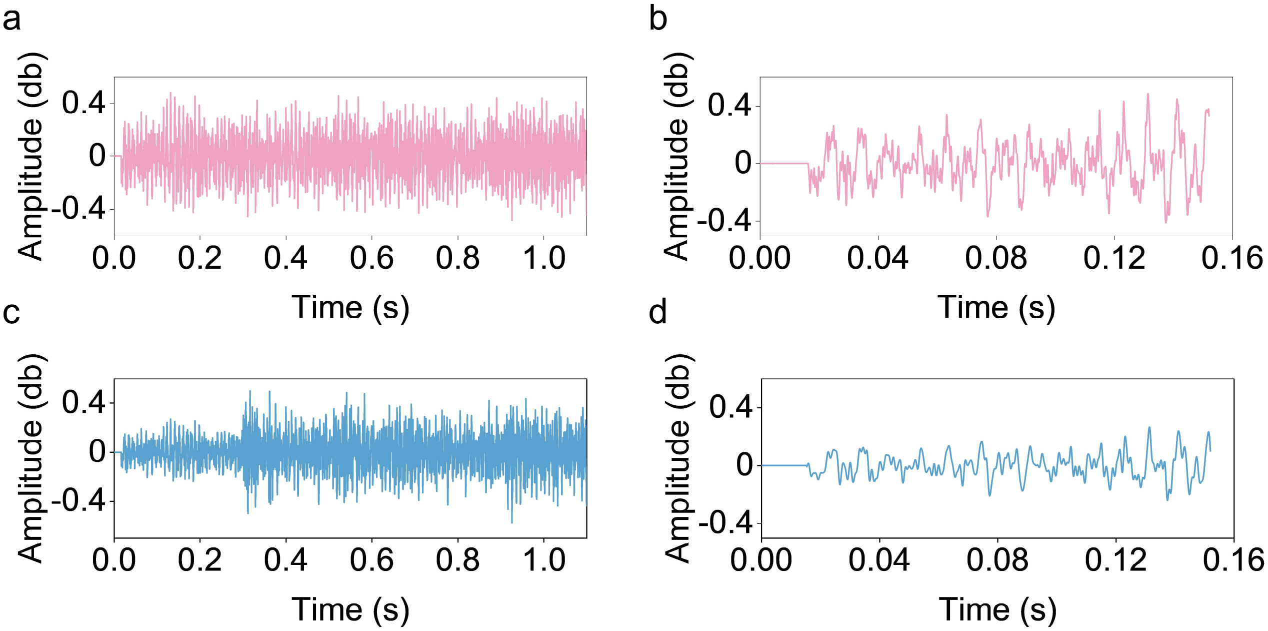

Fig. S30 | Waveform the device under water (blue) and in air (red) (right panel is magnified first 0.16 seconds). a, The waveform of the device playing an audio file in the air. **b**, the first 0.16 seconds of the waveform in panel **a**. **c**, the waveform of the device playing the same audio file under the water (15 mm below the water surface). **d**, the first 0.16 seconds of the waveform in panel **c**.

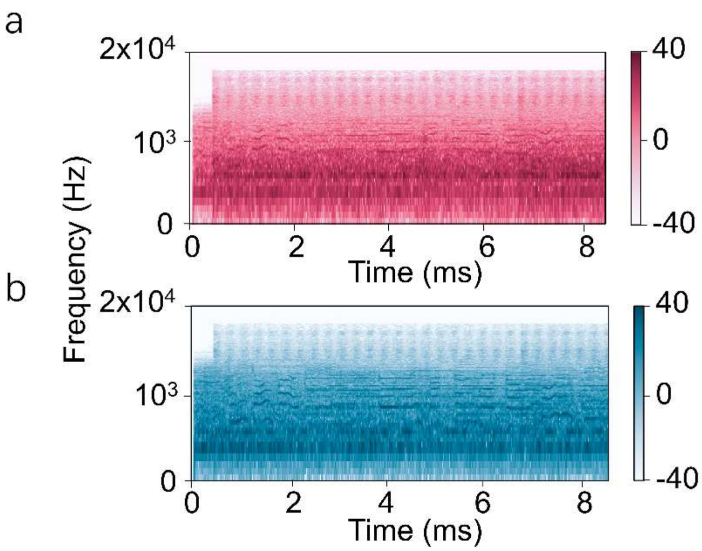

Fig. S31 | Spectrogram of the device under water (blue) and in air (red). a, the spectrum of the device playing the same audio file in the previous figure in air. **b**, the spectrum of the device playing the same audio under water, more noise signal in the high frequency part can be observed.

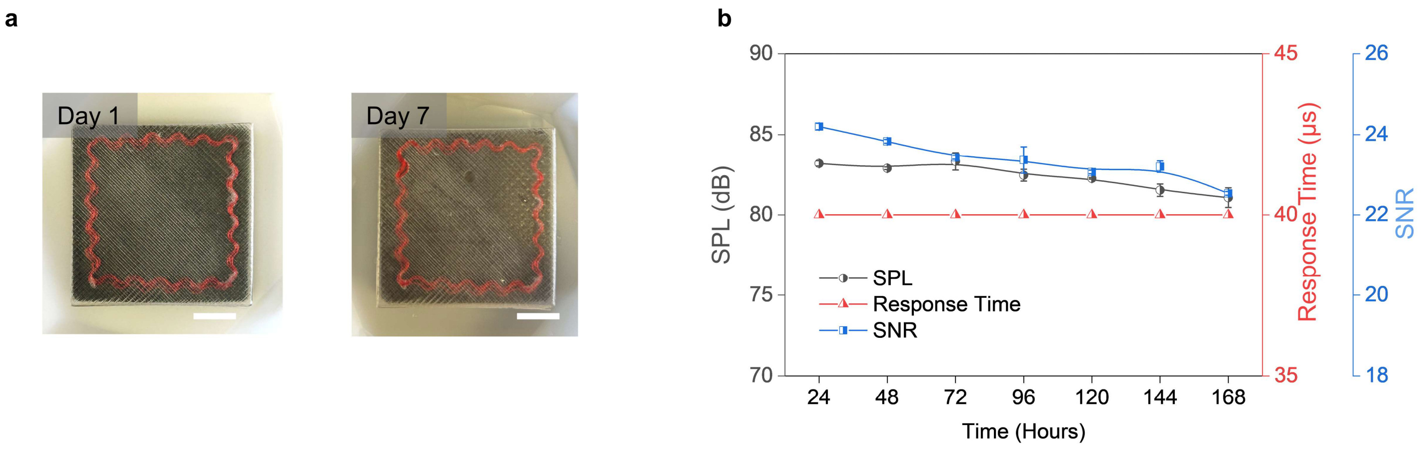

Fig. S32 | Accelerated aging test of the device for waterproofness. **a**, Photo of the device at experimenting day 1 and day 7, scale bar 0.75 cm. **b,** SPL, Response time, and SNR of the device after different soaking hours, a relatively steady output and sensing performance can be observed. Data are presented as mean values ± SD.

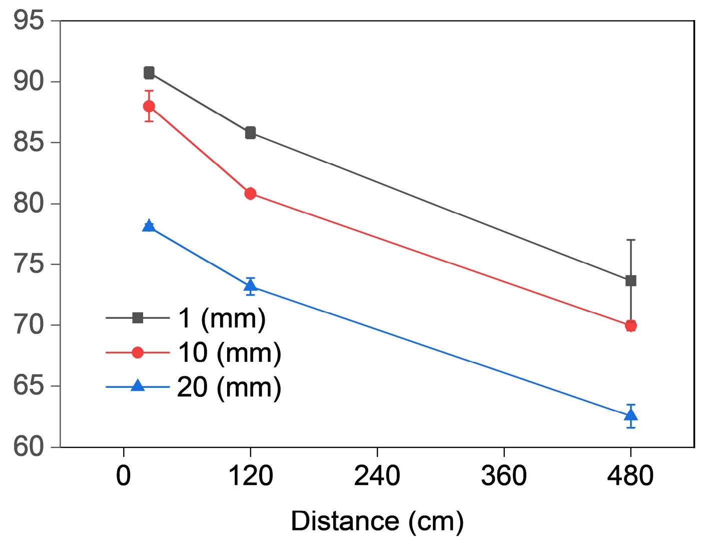

Fig. S33 | Sound pressure level of the device at different depths under water (1, 10, 20 mm), SPL recorded at different distance above the water (1, 5, 20 cm). The relationship between sound pressure level (generated with a tone of 1000 Hz), depth of the device under water and the distance. The deeper the device is put under water, the lower the sound output. A testing of N = 3 times for each scenario is applied. Data are presented as mean values ± SD.

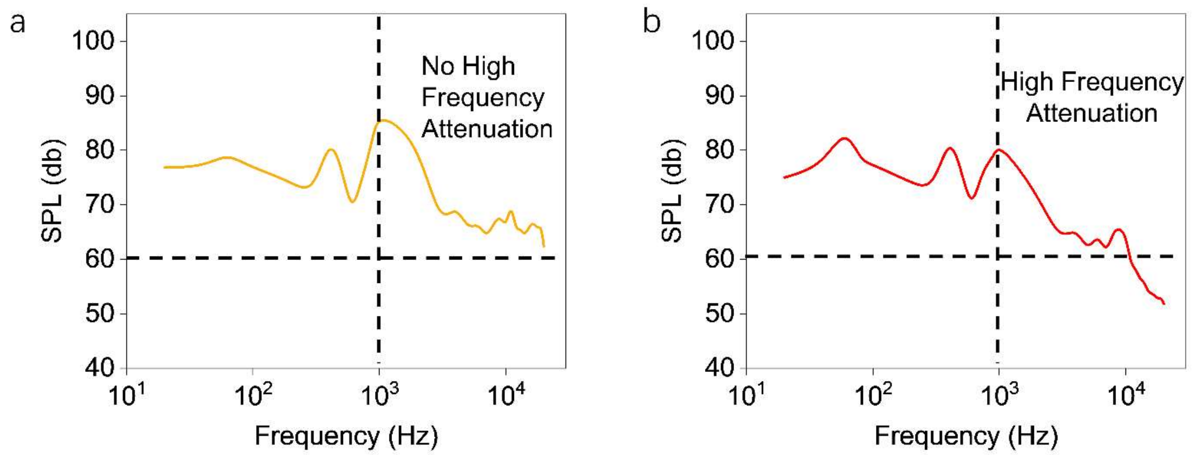

Fig. S34 | Sound pressure level of the device in air (yellow) and under water (red). a, the sound pressure level of the device from 20 to 20,000 Hz in air, no clear high frequency attenuation is observed. **b**, the sound pressure level of the device from 20 to 20,000 Hz underwater, a high frequency attenuation can be observed as the SPL of higher frequency is much lower than the performance in the air.

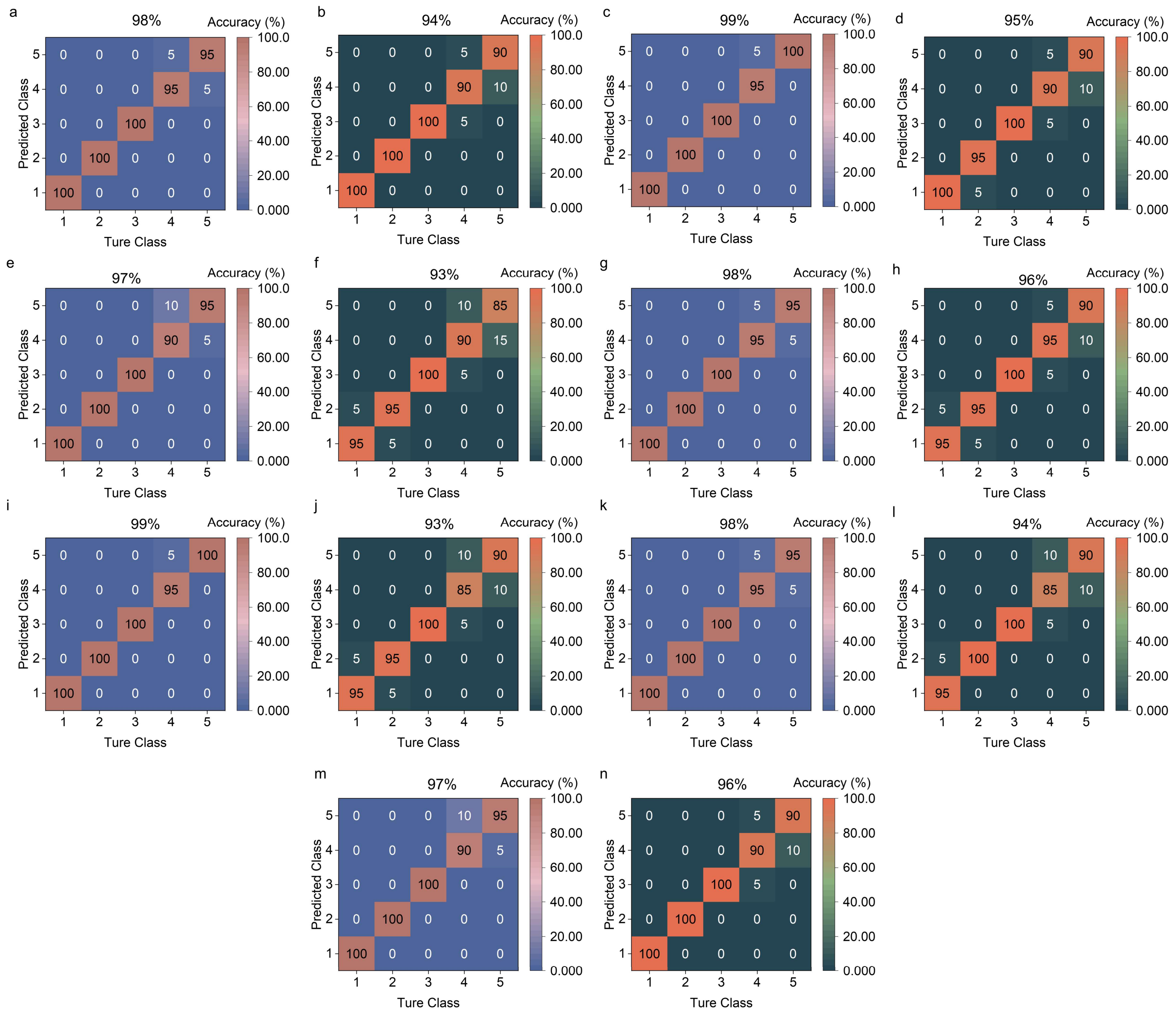

Fig. S35 | Confusion matrix of each participant's validation set (blue) and testing set (green). a, confusion matrix of the First participant's validation set (accuracy of 98%). **b**, confusion matrix of the First participant's testing set (accuracy of 94%) **c**, the confusion matrix of the Second participant's validation set (accuracy of 99%). **d**, confusion matrix of the Second participant's testing set (accuracy of 95%) **e**, confusion matrix of the Third participant's validation set (accuracy of 99%). **f**, confusion matrix of the Third participant's testing set (accuracy of 93%) **g**, confusion matrix of the Forth participant's validation set (accuracy of 98%). **h**, confusion matrix of the Forth participant's testing set (accuracy of 96%) **i**, confusion matrix of the fifth participant's validation set (accuracy of 99%). **j**, confusion matrix of the Fifth participant's testing set (accuracy of 93%) **k**, confusion matrix of the sixth participant's validation set (accuracy of 98%). **l**, confusion matrix of the sixth participant's testing set (accuracy of 94%) **m**, confusion matrix of the Seventh participant's validation set (accuracy of 97%). **n**, confusion matrix of the Second participant's testing set (accuracy of 96%)

Supplementary Notes

Supplementary Notes 1: Fabrication and performance evaluation of the serpentine-shaped coil

The coil's serpentine shape is crafted by wiring coil circles concentrically from the center outward, using a serpentine-shaped mold, as depicted in **Fig. S36**. While the coil is layered sequentially on the xy-plane, its theoretical thickness should equate to the diameter of the copper coil, which is 67 μm. However, during the fabrication process, the wire tends to overlap, even with efforts to flatten it. We assessed the thickness of several coils (a total of six) used in our device and found an average thickness of 147.33 μm (with individual measurements of 144, 156, 141, 139, 159, and 145 μm). This suggests that, on average, two layers of copper wire overlap during fabrication, leading to an increased overall coil thickness.

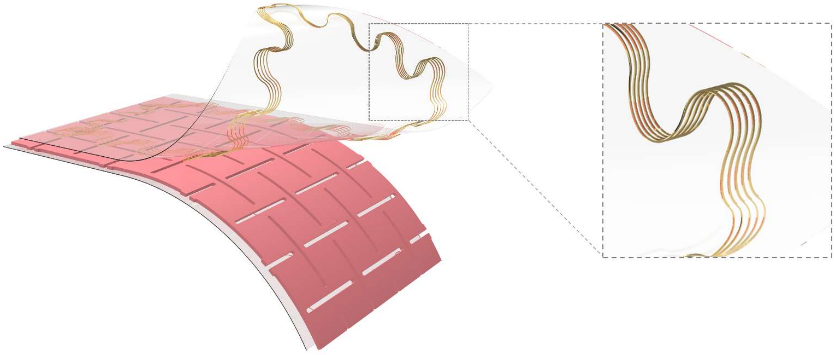

Fig. S36 | Illustration of the MI layer.

In order to further ensure the performance of the coil compared to the non-serpentine-shaped coils, we have conducted the following experiments. In **Fig. S37**a, we tested a serpentine-shaped coil with 20 turns and an outermost side length of **L = 3** cm using a shaker operating at 5 Hz. Aside from a minor fluctuation in signal amplitude at the test's onset, a consistent 5 Hz response signal with an amplitude of approximately 13 μA was observed. Using a similar experimental setup, we tested a non-serpentine-shaped coil with 20 turns. The results, presented in Fig. S37b, show a comparable response waveform with an amplitude of roughly 12 μA. We calculated the Signal-to-Noise ratio for both scenarios, obtaining values of 23.4 dB and 24.6 dB, respectively.

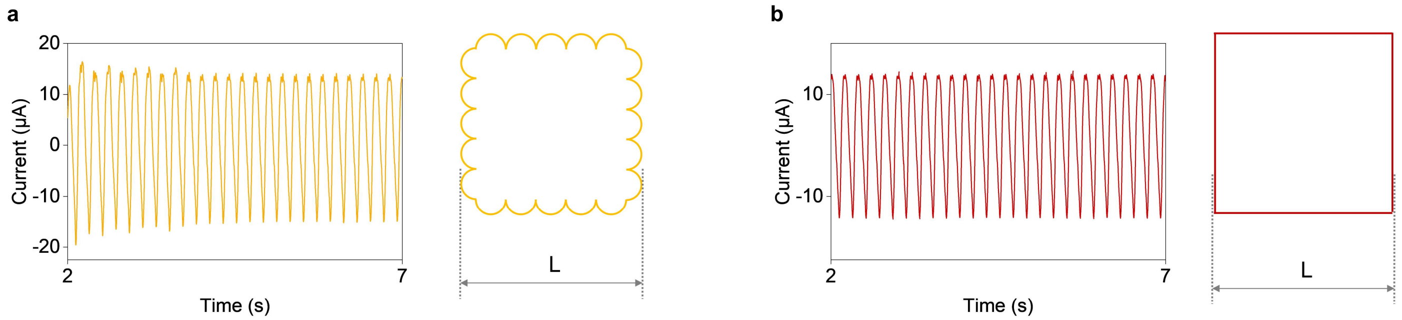

Fig. S37 | Testing of signal generated by two types of coils. a, Signal generated by the serpentine-shaped coil and the illustration of the serpentine-shaped-coil. L = 3 cm in the illustration. **b**, Signal generated by the non-serpentine-shaped coil and its illustration. L = 3 cm in the illustration.

The similarity in performance can be attributed to the current generation principle of our device, which is described by the equation:

$$\varepsilon = -N\frac{\Delta\varphi}{\Delta t}, \varphi = BS$$

Where ε represents the induced electromotive force, N is the number of turns in the coil, φ is the magnetic flux, and t is time, B is the magnetic field intensity, and S is the surface area of the coil.

According to the law of electromagnetic induction, the generated voltage in the circuit is proportional to the coil turns and the rate of change in magnetic flux within the area enclosed by the coil turns. In the cases we examined, the change in magnetic field, ΔB, is produced by the same MC layer that underwent identical deformations, strictly controlled by manipulating the shaker setting (including frequency, amplitude, and strength). In this case, the only variable is S, the coil's area. Given our experimental conditions and the consistent outermost side length, both coils possess nearly identical areas, with minor variations resulting from the serpentine shaping. Consequently, the outputs of both coils are closely matched.

Supplementary Notes 2: Deformation of the device under omnidirectional laryngeal movements

During phonation, omnidirectional deformation occurs, encompassing both in-plane and vertical movements of the skin surface. This is due to the involvement of multiple laryngeal muscle groups, including the extrinsic muscles[7-11] and the platysma muscles[12,13], which are not directly associated with vocal fold control. For example, the sternothyroid muscle, a component of the extrinsic laryngeal muscles, modulates voice pitch by contracting in a direction parallel to the skin surface[7,8,14]. The coordinated contraction and relaxation of these muscles in both vertical and horizontal orientations influence the throat's movement patterns during phonation. This is especially pertinent for patients with voice disorders who are unable to utilize their vocal folds and the associated intrinsic laryngeal muscles. As such, our system's primary objective is to accurately detect these omnidirectional movements of the laryngeal muscle groups.

Deformation within the skin's surface plane (x-y plane) results from the elongation and contraction of muscle bundles during their relaxation and contraction phases, respectively. This in-plane deformation is intuitive, as it directly corresponds to muscle activity. Given that the device is securely adhered to the skin, this deformation acts as a dependent variable, reflecting the direct morphological changes of the muscle bundle.

On the other hand, the z-axis deformation originates from the expansion of the muscle bundle's diameter during contraction. As depicted in **Fig. S38**a, when relaxed, the muscle bundle's diameter (z-axis) reduces, while its length (x-y plane) increases. In contrast, during contraction, as shown in Fig. S38b, the muscle bundle's length decreases, causing a diameter expansion. Fig. S38c and S38d present cross-sectional views of the device adhered to the muscle in these respective states. In its relaxed state, the device conforms closely to the muscle surface, primarily aligning with the x-y plane. However, when the muscle contracts and its diameter enlarge, the device adopts a more contoured alignment on the muscle surface, leading to a z-axis deformation, termed as Dz. Unlike a mere in-plane shift, this results in a "bending" effect on the device. The karigami fabrication technique reduces the device's modulus to a skin-like level, ensuring it deforms in tandem with the body, thereby capturing every mechanical nuance of the laryngeal muscle.

To further determine the relationship between in-plane expansion/ contraction and the z-axis deformation, we have modeled the muscle contraction process in Fig. S38e. Sind the overall shape of the neck does not vary greatly during the phonation process, we have modeled the cross-section of the muscle bundle a semi-circle with a radius R. And the device shown in red is attached to the surface of the circle, the length of the device can be calculated with arc length formula, where θ is the central angle:

$$L = \theta \cdot R$$

The z-axis deformation generated Dz can be calculated by the radius R minus its cosine value of half θ to be:

$$Dz = R - Rcos(\frac{\theta}{2})$$

Since the device is adhesively attached to the skin surface, the relative position between the device and skin does not change during deformation. Under minor deformation, we can set θ as constant, in this case in-plane deformation *dL* can be calculated as:

$$\Delta L = \theta \cdot \Delta R$$

And:

$$\Delta R = \frac{\Delta L}{\theta}, \Delta Dz = \frac{\Delta L}{\theta}(1 - \cos\left(\frac{\theta}{2}\right))$$

So, the deformation in the z-direction linearly dependent on the in-plane expansion/contraction in a minor scale. When the deformation extend increases the θ in not a constant since the circle changes into an oval, the relationship between z-axis deformation and the in-plane expansion/contraction involves a non-constant trigonometric function, thus not linear. While the relationship between Dz and L, θ is not linear, it is nonetheless monotonic, as indicated by the formula provided. This ensures that each distinct laryngeal muscle movement will produce a unique deformation in the device, which in turn generates a unique and identifiable electrical signal for downstream processing. In simpler terms, each specific laryngeal movement is represented by a unique electrical waveform captured by the device's sensing component, thereby guaranteeing the device's sensing accuracy and performance.

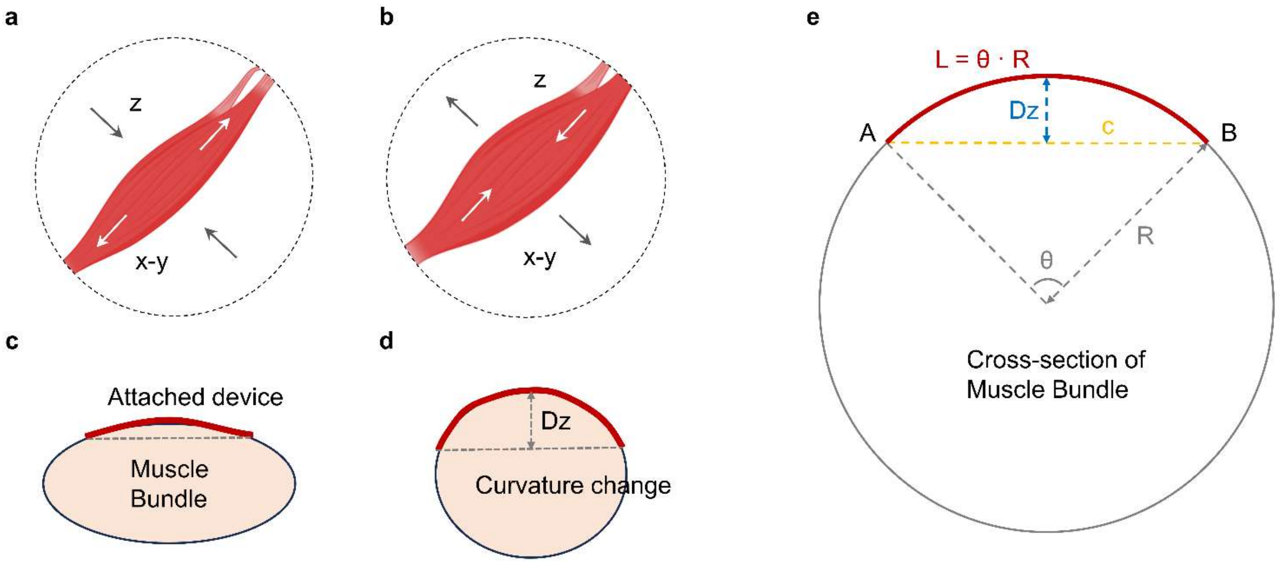

Fig. S38 | The relationship between z-axis deformation and the in-plane expansion/contraction. a, illustration of muscle bundle during relaxation. **b,** illustration of muscle bundle during contraction. **c,** cross-section view of the device attached to the muscle bundle during relaxation. **d,** cross-section view of the device attached to the muscle bundle during contraction. **e,** geometric model of the relationship between z-axis.

Supplementary Notes 3: Examination of the output current and exclusion of the triboelectricity

To prove that there are no triboelectric components in the electric output of the skin-interfaced MEG devices, we identified several key factors necessary for the electrical conversion of TENG: 1. A contact-separation gap must exist between the MEG device and human skin. 2. A single electrode TENG must be grounded to facilitate charge exchange with the ground. Furthermore, the current output of a triboelectric nanogenerator (TENG) typically stays at the μA level, stemming from TENG's capacitive operating mechanism. However, our MEG typically produces a much higher current signal at the mA level. We delve into this comparison further and present detailed experimental results below:

1. **Difference in structure design and operation mode**. A typical MEG holds an all-in-one body design, there is no relative movement among layers. Furthermore, an adhesive layer was applied to prevent any potential sliding between the MEG device and the skin. As depicted in Fig. S39a, our MEG device is securely affixed to the skin with an adhesive layer, which prevents the relative sliding/movement between the MEG device and the skin. The sensing component consists of the MC layer and the MI layer, both of which are fully embedded in the PDMS matrix to form an all-in-one body design.

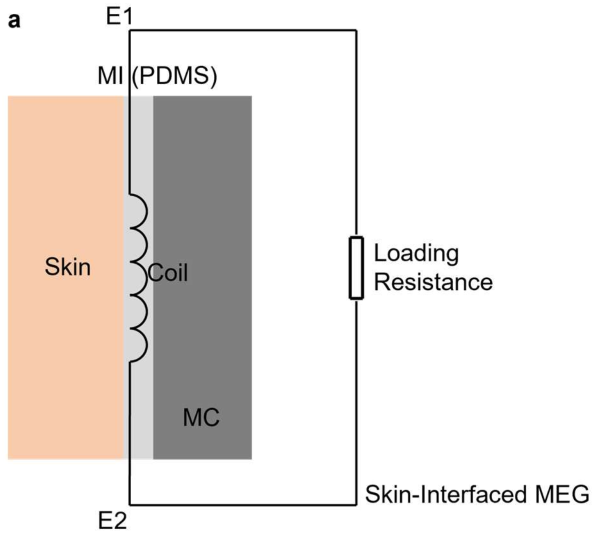

Fig. S39 | Device structure comparison between MEGs and TENGs. a, illustration of device structure of a MEG. **b,** illustration of device structure of a TENG with single electrode mode.

2. **Difference in electrode numbers and connection to loading resistance.** The MEG has two electrode outlets that were connected to the two ends of a loading resistance, while TENG only has one electrode outlet, going through the loading resistance, and then connected to the ground. The electrode connection in the circuit makes our device unable to satisfy the structure of a single-electrode TENG, as illustrated in Fig. S39b. For the single-electrode mode TENG, one circuit end is linked to an electrode that continuously interacts with another material possessing a different electronegativity, while the other end is grounded. This facilitates electron flow to the ground, producing a current. In our design, both electrode ends connect to a current meter or a loading resistance, making it implausible to replicate the single electrode TENG mode.

3. **Order of magnitude difference in electrical output.** The typical current output of a MEG is at the mA level, while the TENG is at the at the μA level. The typical voltage output of a MEG is at the 100-mV level, while the TENG is at the at the 100 V level. The typical inner impedance of a MEG is at the 10 Ω level, while that of a TENG is at the at the MΩ level. In a word, the electric output of TENGs is characteristically high voltage and low current due to their high internal impedance. Conversely, the magnetoelastic effect yields a relatively higher current due to the MEGs' lower internal impedance.

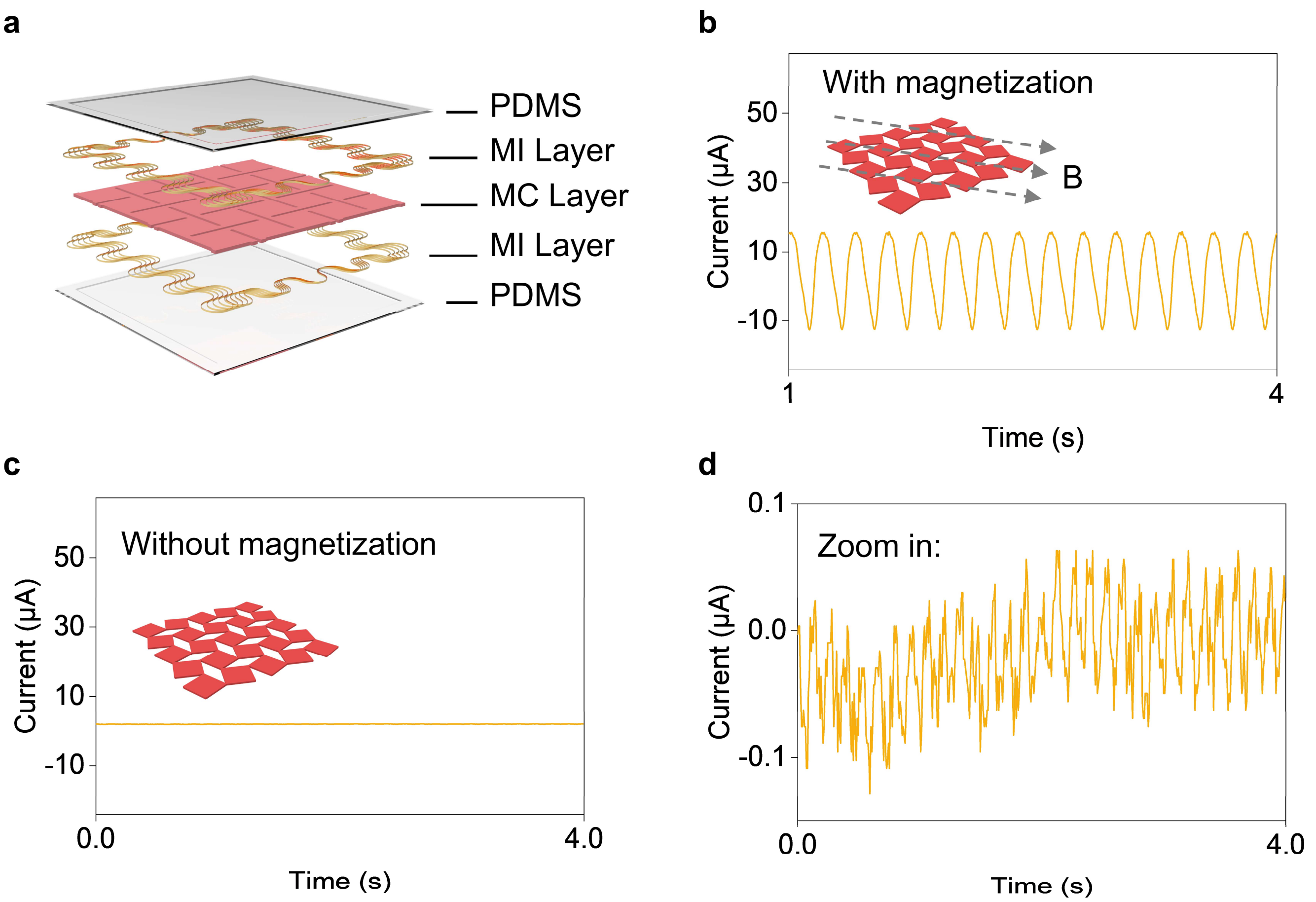

Fig. S40 | Output of MEG with and without magnetization of the MC layer. a, Structure illustration of the MEG. **b,** Output of the device with the magnetization of the MC layer**. c,** Output of the device without the magnetization of the MC layer**. d,** Zoom in on Figure R10c.

In addition, to substantiate this, we conducted experiments detailed as follows: Fig. S40a illustrates that our device operates using two layers - the MC and MI layers. During fabrication, the MC layer undergoes magnetization *via* a pulse magnetizer to induce magnetic flux changes. Initial tests with the standard fabrication process, using a shaker at 5 Hz (Fig. S40b), revealed the device generated a clear signal with an amplitude of 11 μA. In a subsequent test, we employed an identical structure but refrained from magnetizing the MC layer, ensuring no magnetic flux density alterations. Any resulting signal would solely be attributed to potential triboelectric effects. As presented in Fig. S40c, the absence of magnetization in the MC layer resulted in zero electric signal generation. A closer examination in Fig. S40d reveals only a noise signal with an amplitude of approximately 0.05 μA, effectively ruling out the TENG's role in the device's current generation.

Supplementary Table S1 | Weight of different parts of the device

Items	MC Layer	Upper membrane (Actuator)	Upper Coil (Actuator)	Bottom membrane (Sensor)	Bottom Coil (Sensor)	Total (Entire Device)
**Weight (g)**	5.6023	0.7525	0.0534	0.7624	0.0562	7.2268

Supplementary Table S2 | Thickness of the coil with different turn ratios

Sample	20 Turns (μm)	40 Turns (μm)	60 Turns (μm)	80 Turns (μm)	100 Turns (μm)
**1**	144	140	147	154	163
**2**	156	137	155	201	201
**3**	141	146	161	199	270
**4**	139	151	153	160	204
**5**	159	155	198	207	201
**6**	145	151	143	210	211

Supplementary Table S3 | Acoustic performance comparison with other literature

SPL	Distance	Method	Frequency range (Hz)	Driving voltage(V)	Temperature rise (°C)	Modulus	Source
60	50 cm	Magnetoelastic	20-20,000	1.95	Not significant		This work
60	8 cm	Piezoelectric			-	3.79 MPa	1[1]
66	15 cm	Piezoelectric	1000-10,000	25	-	2.6 GPa	2[2]
60	4 cm	Piezoelectric	20-20,000	210	-	40 MPa	3[3]
50	5 cm	Piezoelectric	20-20,000	-	-	0.1Mpa	4[4]
60	5 cm	Thermoacoustic	100-20,000	5	-	2.5GPa	5[5]
60	2.5 cm	Thermoacoustic	20-20,000	-	5.3	-	6[6]

Supplementary References

1. Yan, W. *et al.* Single fibre enables acoustic fabrics *via* nanometre-scale vibrations. *Nature* **603**, 616–623 (2022).
2. Han, J., Lang, J. H. & Bulović, V. An Ultrathin flexible loudspeaker based on a piezoelectric microdome array. *IEEE Trans. Ind. Electron.* **70**, 985–994 (2023).
3. Xu, Z. *et al.* Electrostatic assembly of laminated transparent piezoelectrets for epidermal and implantable electronics. *Nano Energy* **89**, 106450 (2021).
4. Keplinger, C. *et al.* Stretchable, transparent, ionic conductors. *Science* **341**, 984–987 (2013).
5. Yang, Q. *et al.* Mixed-modality speech recognition and interaction using a wearable artificial throat. *Nat. Mach. Intell.* **5**, 169–180 (2023).
6. Tao, L.-Q. *et al.* An intelligent artificial throat with sound-sensing ability based on laser induced graphene. *Nat. Commun.* **8**, 14579 (2017).
7. Ludlow, C. L. Central nervous system control of the laryngeal muscles in humans. Respir Physiol Neurobiol **147**, 205–222 (2005).
8. Honda, K., Hirai, H., Masaki, S. & Shimada, Y. Role of vertical larynx movement and cervical lordosis in F0 control. *Lang Speech* **42**, 401–411 (1999).
9. Kuna, S. T., Smickley, J. S., Vanoye, C. R. & McMillan, T. H. Cricothyroid muscle activity during sleep in normal adult humans. *J. Appl. Physiol.* **76**, 2326–2332 (1994).
10. Titze, I. R., Luschei, E. S. & Hirano, M. Role of the thyroarytenoid muscle in regulation of fundamental frequency. *J. Voice* **3**, 213–224 (1989).
11. Löfqvist, A., McGarr, N. S. & Honda, K. Laryngeal muscles and articulatory control. *JASA* **76**, 951–954 (1984).
12. Baltu, Y. & Baltu, D. Congenital unilateral lower lip palsy with concurrent hypoplasia of the platysma muscle. *Dermatol. Surg.* **44**, 1480 (2018).
13. Galego, J. S., Casas, O. V., Rossato, D., Simões, A. & Balbinot, A. Surface electromyography and electroencephalography processing in dysarthric patients for verbal commands or speaking intention characterization. *Measurement* **175**, 109147 (2021).
14. Burnett, T. A. & Larson, C. R. Early pitch-shift response is active in both steady and dynamic voice pitch control. *JASA* **112**, 1058–1063 (2002).
