# Supplementary material for: Speaking without vocal folds using a machine-learning-assisted wearable sensing-actuation system
Source: Nat Commun. 2024 Mar 12;15:1873. doi: 10.1038/s41467-024-45915-7 (PMC10933441; doi:10.1038/s41467-024-45915-7)
Supplement: Supplementary file 3 — Reporting Summary [file 41467_2024_45915_MOESM3_ESM.pdf]

Corresponding author(s): Jun Chen

Last updated by author(s): Dec 15, 2023

Reporting Summary

Nature Portfolio wishes to improve the reproducibility of the work that we publish. This form provides structure for consistency and transparency in reporting. For further information on Nature Portfolio policies, see our Editorial Policies and the Editorial Policy Checklist.

Statistics

For all statistical analyses, confirm that the following items are present in the figure legend, table legend, main text, or Methods section.

n/a	Confirmed	
☐	☒	The exact sample size (*n*) for each experimental group/condition, given as a discrete number and unit of measurement
☐	☒	A statement on whether measurements were taken from distinct samples or whether the same sample was measured repeatedly
☒	☐	The statistical test(s) used AND whether they are one- or two-sided<br>*Only common tests should be described solely by name; describe more complex techniques in the Methods section.*
☐	☒	A description of all covariates tested
☐	☒	A description of any assumptions or corrections, such as tests of normality and adjustment for multiple comparisons
☐	☒	A full description of the statistical parameters including central tendency (e.g. means) or other basic estimates (e.g. regression coefficient) AND variation (e.g. standard deviation) or associated estimates of uncertainty (e.g. confidence intervals)
☒	☐	For null hypothesis testing, the test statistic (e.g. *F*, *t*, *r*) with confidence intervals, effect sizes, degrees of freedom and *P* value noted<br>*Give P values as exact values whenever suitable.*
☒	☐	For Bayesian analysis, information on the choice of priors and Markov chain Monte Carlo settings
☒	☐	For hierarchical and complex designs, identification of the appropriate level for tests and full reporting of outcomes
☒	☐	Estimates of effect sizes (e.g. Cohen's *d*, Pearson's *r*), indicating how they were calculated

Our web collection on statistics for biologists contains articles on many of the points above.

Software and code

Policy information about availability of computer code

Data collection	No custom code for data collection that was central to the conclusions was used in this study.
Data analysis	No custom code or mathematical algorithm that was central to the conclusions was used in this study.

For manuscripts utilizing custom algorithms or software that are central to the research but not yet described in published literature, software must be made available to editors and reviewers. We strongly encourage code deposition in a community repository (e.g. GitHub). See the Nature Portfolio guidelines for submitting code & software for further information.

Data

Policy information about availability of data

All manuscripts must include a data availability statement. This statement should provide the following information, where applicable:

- Accession codes, unique identifiers, or web links for publicly available datasets
- A description of any restrictions on data availability
- For clinical datasets or third party data, please ensure that the statement adheres to our policy

All data supporting the findings of this study are available within the article and its supplementary files. Any additional requests for information can be directed to, and will be fulfilled by, the corresponding authors. Source data are provided with this paper at DOI: 10.6084/m9.figshare.24784107.

Human research participants

Policy information about studies involving human research participants and Sex and Gender in Research.

Reporting on sex and gender	In total of 8 participants were recruited in the experiment testing device performance through questionnaire among UCLA students. Among which 4 participants are female and 4 are male. The gender information is obtained based on self-reporting method of the participant. Gender and other biographical information are not relevant to the human study conducted in our experiment.
Population characteristics	Human participants in this study are UCLA undergrad students with a age range of 18-22 years old. No population characteristics are relevant to the experiment and result in this study.
Recruitment	Participants are recruited with questionnaire send out through posters on the UCLA campus. Participants will contact the authors of this paper through email and conduct following experiments.
Ethics oversight	All participating subjects of this research are informed, and written consent of all participants were obtained before the study. The speaking without vocal folds using machine-learning-assisted wearable sensing-actuation system was conducted in compliance with all the ethical regulations under a protocol (ID: 20-001882) that was approved by the Institutional Review Board (IRB) at University of California, Los Angeles.

Note that full information on the approval of the study protocol must also be provided in the manuscript.

Field-specific reporting

Please select the one below that is the best fit for your research. If you are not sure, read the appropriate sections before making your selection.

☒ Life sciences ☐ Behavioural & social sciences ☐ Ecological, evolutionary & environmental sciences

For a reference copy of the document with all sections, see nature.com/documents/nr-reporting-summary-flat.pdf

Life sciences study design

All studies must disclose on these points even when the disclosure is negative.

Sample size	Sample size used in this study in N = 8. Where we recruited 8 participants in the testing of our device. The sample size is chosen to prove the device is able to function on different people. The research subject is the device rather than human participants. So 8 was chosen as the sample size where it is sufficient to prove the consistency of the performance of the device on 8 different people
Data exclusions	No data were excluded from the analysis
Replication	We have conducted three repeats of the device testing in our study. The repeat is chosen because the testing subject is the device rather the effect of the device on human participants. The performance of the device is relatively stable compared to biological parameters so 3 repeats under the same testing participant and environment is enough to show the performance.
Randomization	The experiments were not randomized.
Blinding	The Investigators were not blinded to allocation during experiments and outcome assessment.

Reporting for specific materials, systems and methods

We require information from authors about some types of materials, experimental systems and methods used in many studies. Here, indicate whether each material, system or method listed is relevant to your study. If you are not sure if a list item applies to your research, read the appropriate section before selecting a response.

Materials & experimental systems

n/a	Involved in the study	
☒	☐	Antibodies
☒	☐	Eukaryotic cell lines
☒	☐	Palaeontology and archaeology
☒	☐	Animals and other organisms
☒	☐	Clinical data
☒	☐	Dual use research of concern

Methods

n/a	Involved in the study	
☒	☐	ChIP-seq
☒	☐	Flow cytometry
☒	☐	MRI-based neuroimaging
